# Engineering prokaryotic channels for control of mammalian tissue excitability

**DOI:** 10.1038/ncomms13132

**Published:** 2016-10-18

**Authors:** Hung X. Nguyen, Robert D. Kirkton, Nenad Bursac

**Affiliations:** 1 Department of Biomedical Engineering, Duke University, 101 Science Drive, Room 1427, Fitzpatrick CIEMAS, Durham, North Carolina 27708, USA

## Abstract

The ability to directly enhance electrical excitability of human cells is hampered by the lack of methods to efficiently overexpress large mammalian voltage-gated sodium channels (VGSC). Here we describe the use of small prokaryotic sodium channels (BacNa_v_) to create *de novo* excitable human tissues and augment impaired action potential conduction *in vitro*. Lentiviral co-expression of specific BacNa_v_ orthologues, an inward-rectifying potassium channel, and connexin-43 in primary human fibroblasts from the heart, skin or brain yields actively conducting cells with customizable electrophysiological phenotypes. Engineered fibroblasts (‘E-Fibs') retain stable functional properties following extensive subculture or differentiation into myofibroblasts and rescue conduction slowing in an *in vitro* model of cardiac interstitial fibrosis. Co-expression of engineered BacNa_v_ with endogenous mammalian VGSCs enhances action potential conduction and prevents conduction failure during depolarization by elevated extracellular K^+^, decoupling or ischaemia. These studies establish the utility of engineered BacNa_v_ channels for induction, control and recovery of mammalian tissue excitability.

## Abstract (web summary)

Restoring lost excitability of injured tissue is a paramount of regenerative medicine. By using a combined expression of bacterial voltage-gated Na^+^ channel, Kir2.1, and connexin-43 in non-excitable human fibroblasts, here the authors generate excitable cells that rescue action potential conduction in an *in vitro* model of cardiac fibrosis.

## Graphical abstract

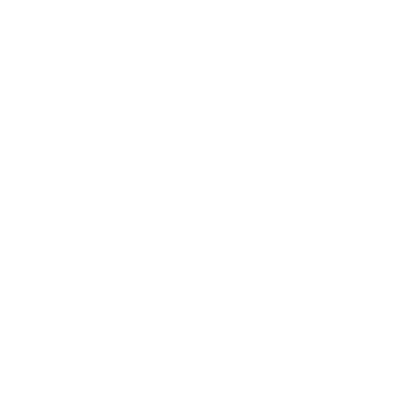

Voltage-gated sodium channels (VGSCs) exist in all animal organisms and govern processes fundamental to life including neuronal communication, muscle contractions or hormonal release^1^. In electrically excitable tissues, VGSCs enable firing and spread of action potentials (APs) and their loss-of-function mutations^2^ cause a variety of neuronal^3^,^4^,^5^,^6^, cardiac^7^,^8^ and skeletal muscle^9^,^10^ disorders. Similarly, acute tissue injuries resulting in permanent excitability loss (for example, stroke, spinal cord injury and heart attack) are associated with long-term disability and death. In addition to excitable tissues, the VGSCs are expressed in many unexcitable cells including macrophages, microglia and islet β-cells, where they play important roles in phagocytosis, migration, insulin secretion and other cellular processes^11^. The ion-conducting α-subunits of mammalian VGSCs have four structurally homologous domains, each consisting of ∼300 amino acids^12^. These four domains are simultaneously transcribed and translated from a single, relatively large (>6 kb) gene. This large gene size precludes stable and efficient expression of mammalian VGSCs in primary cells, a strategy that could potentially lead to new remedies for impaired tissue excitability.

The recent discovery of a large bacterial VGSC family (BacNa_v_) has provided novel insights into the structure and biophysical properties of eukaryotic VGSCs^13^,^14^,^15^,^16^,^17^. In contrast to their eukaryotic counterparts, BacNa_v_^13^ consist of four identical domains, individually transcribed and translated from single genes of only ∼900 bp in size. Although the small size of BacNa_v_ facilitates their overexpression and purification for crystallography studies^18^, it is plausible that *BacNa*_*v*_ genes could be efficiently packaged into viral vectors, either alone or with other ion channel genes, and used to stably introduce or modify electrical excitability of mammalian tissues. Furthermore, utilization of BacNa_v_ orthologues with diverse biophysical properties^13^,^14^,^15^,^16^,^19^,^20^, combined with targeted channel mutations, may allow custom engineering of cellular electrophysiological phenotypes tailored to specific tissue types, diseases and individuals.

Here we combined electrophysiological, computational and genetic engineering techniques to establish a versatile BacNa_v_-based platform that enables stable conversion of unexcitable primary human fibroblasts into AP conducting cells that can successfully recover conduction slowing in an *in vitro* model of cardiac interstitial fibrosis. Moreover, we demonstrate that direct expression of engineered BacNa_v_ channels in mammalian excitable cells can enhance their excitability and prevent loss of AP conduction in pathological conditions when endogenous Na^+^ channels fail to activate.

## Results

### Genetic engineering of actively conducting human fibroblasts

**Figure 1:**
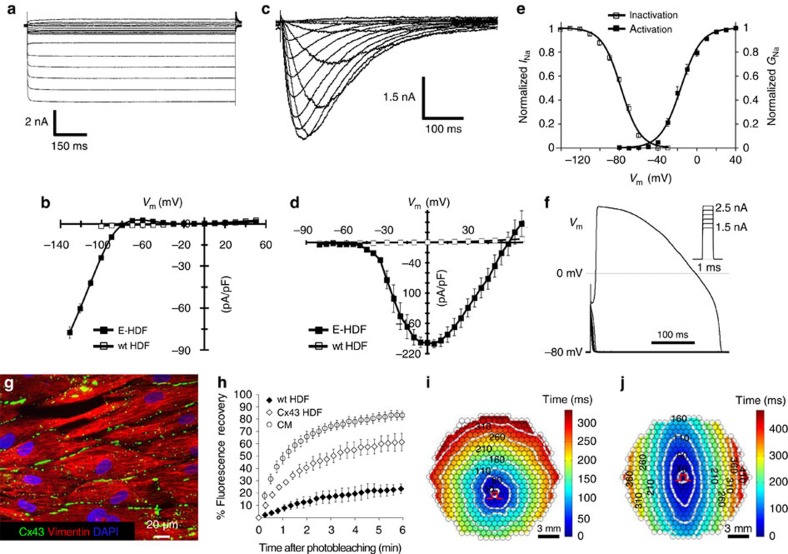
Generation of actively conducting human E-Fibs. (**a**) Voltage-clamp recording of *I*_K1_ in E-HDFs. (**b**) Steady-state *I*_K1_–*V* curves in E-HDF and wt (unexcitable) HDF. (**c**–**e**) Recording of *I*_Na_ (**c**), peak *I*_Na_–*V* curve (**d**) and voltage dependence of *I*_Na_ activation and steady-state inactivation (**e**) in E-HDFs expressing Na_v_RosD G217A. In **b**,**d**,**e**: *n*=8–10. Data in **a**–**e** recorded at 25 °C. (**f**) All-or-none AP response in E-HDFs induced by current pulses (*I*_m_) of increasing amplitude. (**g**,**h**) Stable overexpression of Cx43 in E-HDFs (**g**) results in formation of functional gap junctions, shown by increased recovery of fluorescence after photobleaching, albeit at a slower rate than that of neonatal rat cardiomyocytes (CM) (**h,** *n*=6). (**i**,**j**) Representative isochrone maps of AP conduction in electrically stimulated isotropic (**i**) and anisotropic (**j**) monolayers of E-HDFs stably co-expressing Kir2.1, Na_v_RosD G217A and Cx43. Pulse signs indicate position of stimulating electrode. Circles denote 504 recording sites. All electrophysiological data obtained at 37 °C, unless otherwise specified. Error bars indicate s.e.m.

To stably convert unexcitable primary fibroblasts into actively conducting cells (named engineered fibroblasts or ‘E-Fibs'), we first explored a BacNa_v_ variant from *Roseobacter denitrificans*, Na_v_RosD G217A, shown in our preliminary tests to be capable of generating AP propagation when transfected in a monolayer of HEK293 cells stably expressing Kir2.1 and connexin-43 (Cx43). We generated a high-titre bi-cistronic lentivirus pRRL-CMV-RosDG217A-P2A-eGFP-Kir2.1 encoding both Na_v_RosD G217A and inward-rectifier potassium channel Kir2.1 (gene *KCNJ2*) fused with enhanced green fluorescent protein (eGFP) (eGFP-Kir2.1), separated by the self-cleaving peptide P2A. Adult human dermal fibroblasts (HDFs) transduced with this virus stably expressed inward-rectifier K^+^ current, *I*_K1_ (Fig. 1a,b), and bacterial Na^+^ current, *I*_NaRosD G217A_ (Fig. 1c,d), at much higher levels compared with endogenous currents, and exhibited a significantly hyperpolarized resting membrane potential of −78.4±0.2 mV (*n*=6) compared with that of wild-type (wt)-HDFs (−23.3±2.9 mV; *n*=5). The resulting voltage dependencies of activation and inactivation of Na_v_RosD G217A (Fig. 1e) in transduced HDFs were similar to those previously reported in transfected HEK293s (ref. 15). Importantly, transduced HDFs became electrically excitable, whereby injection of 1 ms current pulses of increasing amplitude elicited reproducible firing of an ‘all-or-none' AP (Fig. 1f). These results demonstrated the ability of BacNa_v_ to yield creation of *de novo* electrically excitable cells.

To enable electrical coupling in excitable HDFs, we co-transduced them with a pRRL-CMV-Cx43-P2A-mCherry lentivirus. Transduced cells robustly expressed membrane-bound Cx43 gap junctions (Fig. 1g), which resulted in improved intercellular coupling compared with wt-HDFs as revealed by fluorescence recovery after photobleaching^21^, albeit not to the same level as measured in neonatal rat cardiomyocytes (Fig. 1h). These engineered HDFs (E-HDFs) were then cultured at confluence in 2 cm diameter monolayers in a random or aligned configuration (Supplementary Fig. 1). When electrically stimulated by a bipolar platinum electrode, randomly oriented E-HDFs supported circular AP spread at an average conduction velocity (CV) of 3.63±0.23 cm s^−1^ (Fig. 1i and Supplementary Video 1). As expected, aligned E-HDFs exhibited elliptical (anisotropic) AP spread with higher CVs along (3.86±0.14 cm s^−1^, *n*=5) versus across (2.07±0.19 cm s^−1^, *n*=5) cell orientation (Fig. 1j and Supplementary Video 1). Although the above approach enabled AP propagation in initially unexcitable human fibroblasts, CVs were low, which could be at least in part attributed to the relatively slow activation kinetics of Na_v_RosD G217A.

### Improved E-Fib electrical properties with NavSheP mutants

**Figure 2:**
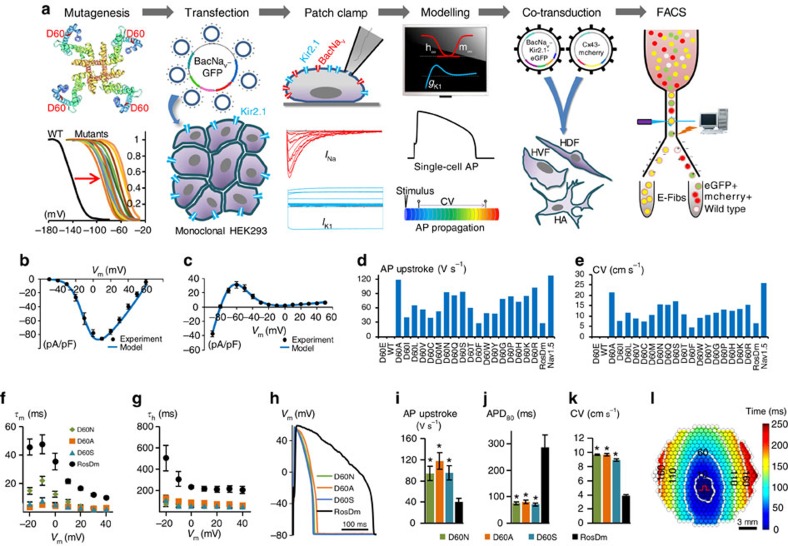
Customization of E-Fib phenotype via mutagenesis. (**a**) BacNa_v_-based strategy for E-Fib customization. (**b**) Experimental (*n*=8) and modelling peak *I*_Na_–*V* curves. Cell membrane was held at −80 mV before stepping the voltage from −50 to 60 mV in 10 mV increments. (**c**) Experimental (*n*=21) and modelling steady-state *I*_K_–*V* curves. Cell membrane was held at −40 mV before stepping the voltage from −90 to 50 mV in 10 mV increments. (**d**,**e**) AP initiation and conduction characteristics of BacNa_v_ mutant expressing E-HDFs obtained by computational modeling: upstroke velocity (**d**) and CV (**e**). For modelling, parameters derived from voltage-clamp measurements at 25 °C were scaled for the temperature of 37 °C. Highly hyperpolarized inactivation of WT and D60E channels prevented AP initiation. (**f**,**g**) Time constants of activation (**f**) and inactivation (**g**) of selected Na_v_SheP D60X mutants compared with Na_v_RosD G217A (RosDm), recorded in E-HEK293 cells at 25 °C (*n*=5). (**h**–**j**) When co-expressed with Kir2.1 in E-HEK293 cells, Na_v_SheP mutants give rise to APs (**h**) with faster upstroke (**i**; *n*=10–18) and shorter duration (**j**; *n*=10–18) than Na_v_RosD G217A (*n*=11 for **i**,**j**). (**k**) Na_v_SheP D60X expressions in anisotropic E-HDF monolayers yield faster CV than RosDm expression (*n*=5–10). (**l**) Representative isochrone map of AP conduction in an electrically stimulated anisotropic monolayer of E-HDFs stably co-expressing Kir2.1, Na_v_SheP D60N and Cx43 (compare also with Fig. 1j). *P<0.001 versus RosDm (**i**–**k**). All electrophysiological data obtained at 37 °C, unless otherwise specified. Error bars indicate s.e.m; statistical significance was determined by one-way analysis of variance, followed by Tukey's *post-hoc* test to calculate *P*-values.

To augment the excitability and conduction of E-HDFs, we developed a combined computational and experimental strategy to screen and identify BacNa_v_ variants with more favourable properties (Fig. 2a). We set to explore the BacNa_v_ orthologue from *Shewanella putrefaciens*, Na_v_SheP, for its fast current kinetics^15^. As the wt Na_v_SheP channel has a highly hyperpolarized voltage dependency of steady-state inactivation, it does not yield AP firing when co-expressed with Kir2.1. We thus proceeded to shift the activation and inactivation curves of Na_v_SheP to more depolarized voltages based on the previous studies of specific residues in NaChBac channels^22^ and significant homology between different BacNa_v_ orthologues (Supplementary Fig. 2); we created a library of Na_v_SheP mutants where either the D60 or E43 residue was substituted with other standard amino acids. These 38 Na_v_SheP mutants were transiently expressed in a monoclonal HEK293 line stably expressing Kir2.1 and the resulting sodium current was recorded by voltage clamp. The E43 mutants exhibited highly depolarized voltage dependence of activation (Supplementary Table 1); therefore, we focused on detailed characterization of D60 mutants. The D60 mutant channels exhibited a wide range of steady-state and kinetic parameters (Supplementary Fig. 3 and Supplementary Table 1) and predominantly exhibited fast kinetics compared with the Na_v_RosD channel (Supplementary Fig. 3c,d and Supplementary Table 1). Based on the recorded parameters, we applied computational modelling to infer AP upstroke, threshold, duration and CV that would result from the same expression level of different Na_v_SheP mutants in an electrically coupled, Kir2.1 HEK293 line (Fig. 2b–e and Supplementary Fig. 4) and constructed Na_v_SheP D60X-Kir2.1 lentiviruses for three D60X mutants (D60A, D60N and D60S) that exhibited the steepest AP upstrokes and highest CVs among different BacNa_v_ models. All three mutants exhibited significantly faster kinetics compared with Na_v_RosD G217A in transduced cells (Fig. 2f,g), which allowed generation of APs with shorter duration and sharper upstroke velocity (Fig. 2h–j). Co-transduction of these viruses with Cx43 in HDFs yielded a 2.5-fold improvement in CV compared with the use of Na_v_RosD (Fig. 2k,l and Supplementary Video 2). This demonstrated the potential for combining BacNa_v_ mutagenesis and computational modelling, to engineer primary human fibroblasts with desired electrophysiological phenotypes.

### Fine-tuning of E-Fib electrical properties via FACS

**Figure 3:**
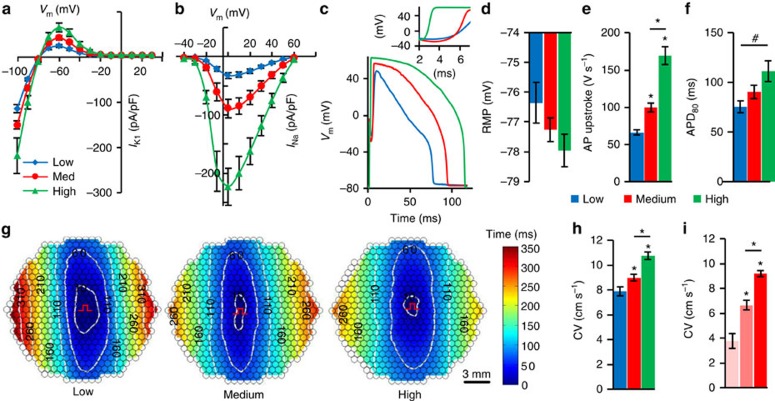
Tuning electrical properties of E-Fibs via FACS. (**a**,**b**) Steady-state *I*_K1_–*V* curves (**a**; *n*=8–12) and peak *I*_Na_–*V* curve (**b**; *n*=8–12) in three groups (blue, red and green denoting low, medium and high eGFP-expressing cells, respectively), measured at 25 °C. (**c**) Representative AP traces with inset showing AP upstrokes in the three eGFP-sorted E-HDF groups. (**d**–**f**) Resting membrane potential (RMP, **d**), maximum AP upstroke velocity (**e**) and APD_80_ (**f**) in the three eGFP-sorted E-HDF groups (*n*=14–18). (**g**,**h**) Representative isochrone maps of AP conduction in electrically stimulated anisotropic monolayers of E-HDFs (**g**) showing that cells with higher eGFP expression had faster CV (**h**; *n*=6–10). All E-HDFs were first sorted for high Cx43-mCherry level to ensure strong intercellular coupling. Pulse signs indicate location of stimulating electrode. Circles denote 504 recording sites. (**i**) CV in electrically stimulated monolayers of three mCherry-sorted E-HDF groups (left, middle and right bars denoting low, medium and high mCherry-expressing cells, respectively; *n*=5). #*P*<0.01 in **f**; *P<0.001 versus low eGFP group in **e**,**h** or versus low or medium mCherry group in **i**. All electrophysiological data obtained at 37 °C, unless otherwise specified. Error bars indicate s.e.m; statistical significance was determined by one-way analysis of variance, followed by Tukey's *post-hoc* test to calculate *P*-values.

The use of fluorescence reporters in multi-cistronic lentiviruses (for example, pRRL-CMV-SheP D60N-P2A-eGFP-Kir2.1) allowed us to further vary *I*_NaSheP_ and *I*_K1_ levels, and characterize their functional effects on E-HDFs. Using fluorescence-activated cell sorting (FACS), we sorted E-HDFs into three populations with low, medium and high GFP expression levels (Supplementary Fig. 5a), which yielded corresponding low, medium and high levels of *I*_K1_ (Fig. 3a) and *I*_NaSheP_ (Fig. 3b), and distinct changes in AP shape (Fig. 3c). Specifically, a trend towards more hyperpolarized resting membrane potential (Fig. 3d, one-way analysis of variance, *P*=0.15) and the significantly higher maximum upstroke velocity (Fig. 3e) could be attributed to higher *I*_K1_ and *I*_NaSheP_, respectively, whereas the increased APD (Fig. 3f) resulted from net depolarizing effect of increased *I*_K1_ and *I*_NaSheP_ on membrane repolarization. Although higher AP upstroke velocities expectedly yielded higher CVs (Fig. 3g,h), this effect was moderate, which we hypothesized was due to simultaneous increase in *I*_K1_ along with *I*_NaSheP_. We thus modelled concurrent increase in *I*_K1_ and *I*_NaSheP_ to simulate the experiment (model 1; Supplementary Fig. 6) and compared resulting CVs with those caused by an increase in *I*_NaSheP_ alone (model 2; Supplementary Fig. 6). Despite the same AP upstroke velocities in both models (Supplementary Fig. 6a), CV in model 1 increased less (Supplementary Fig. 6b), because more depolarizing current was needed to overcome high *I*_K1_, reach threshold and elicit AP (Supplementary Fig. 6c). Examining a wide range of channel conductances (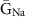 and 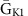) further revealed that although threshold current required to elicit AP is primarily sensitive to level of *I*_K1_ (Supplementary Fig. 6d), AP upstroke velocity is mainly dependent on *I*_NaSheP_ (Supplementary Fig. 6e), with these two currents inducing opposite effects on CV (Supplementary Fig. 6f). Furthermore, as E-HDFs were also transduced with pRRL-CMV-Cx43-P2A-mCherry lentivirus, sorting cells based on mCherry fluorescence level (Supplementary Fig. 5c) allowed us to vary Cx43 expression and gap junctional coupling of engineered cells. Optical mapping revealed that E-HDFs with higher mCherry/Cx43 expression supported faster AP propagation (Fig. 3i), whereas contrary to eGFP sorting (Supplementary Fig. 5b), mCherry sorting had no effect on APD_80_ (Supplementary Fig. 5d). Simulation results further revealed that intercellular coupling strength does not influence the relative effects of varying *I*_K1_ and *I*_NaSheP_ on CV (Supplementary Fig. 6g).

### Robustness of E-Fib electrical phenotype

**Figure 4:**
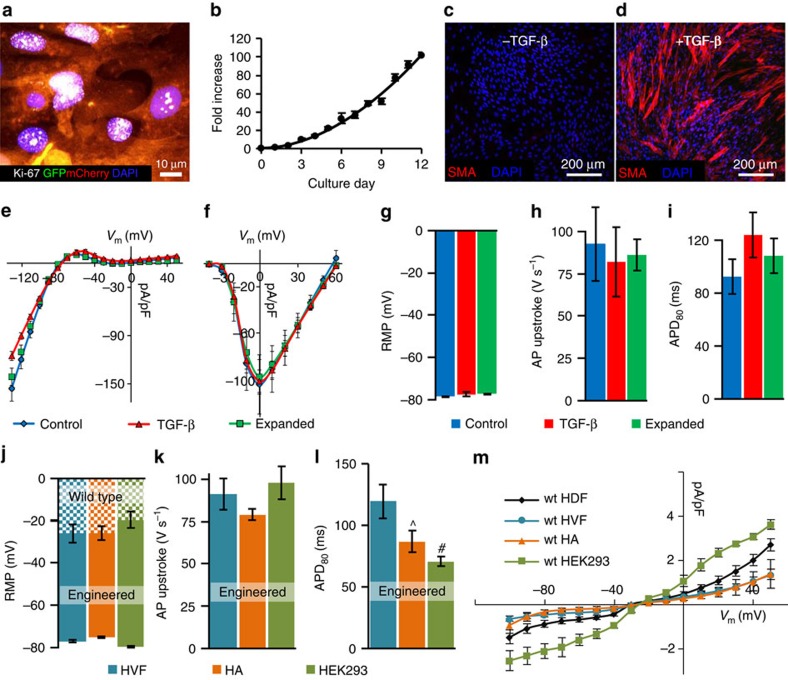
Stability and robustness of E-Fib phenotype. (**a**,**b**) E-HDFs transduced with Na_v_SheP D60N-P2A-eGFP-Kir2.1 and Cx43-P2A-mCherry lentiviruses retain proliferative potential as shown by Ki-67-positive staining (**a**) after ∼100-fold expansion (**b**), 4 weeks post-lentiviral transduction. Growth curve is fit to a power function. (**c**,**d**) Conversion of smooth muscle actin (SMA)^−^ E-HDFs (**c**) to SMA^+^ myofibroblasts after transforming growth factor (TGF)-β treatment (**d**). (**e**–**i**) Steady-state *I*_K1_–*V* (**e**) and peak *I*_Na_–*V* (**f**) curves (recorded at 25 °C) as well as resting membrane potential (RMP, **g**), APD_80_ (**h**) and AP upstroke velocity (**i**) do not differ among control, expanded or TGF-β-treated E-HDFs. (*n*=5–9). (**j**–**l**) RMP (**j**), maximum AP upstroke velocity (**k**) and APD_80_ (**l**) in different E-Fibs (E-HVF, human ventricular fibroblasts; E-HA, human astrocytes) transduced with the Na_v_SheP D60N-P2A-eGFP-Kir2.1 lentivirus (*n*=6–8). (**m**) Steady-state *I*–*V* curves in different wt Fibs (*n*=4–6). ^*P*<0.05, #*P*<0.01 versus HVF in **l**. All electrophysiological data obtained at 37 °C, unless otherwise specified. Error bars indicate s.e.m; statistical significance was determined by one-way analysis of variance, followed by Tukey's *post-hoc* test to calculate *P*-values.

To test whether E-HDFs can maintain stable electrophysiological phenotype under various perturbations, we expanded them 500-fold over a 4-week period, either directly or after 2 freeze–thaw cycles. In both cases, expanded E-HDFs retained proliferative potential (Fig. 4a) and were able to undergo additional rounds of expansion (for example, 100-fold, 4 passages; Fig. 4b). In addition, as fibroblasts can differentiate into smooth muscle actin expressing myofibroblasts during wound healing and tissue repair^23^, we treated the expanded E-HDFs with 10 ng ml^−1^ transforming growth factor-β1 for 7 days, to induce their conversion to myofibroblasts (Fig. 4c,d). Subsequent electrophysiological analysis revealed that despite significant cell expansion or myofibroblast differentiation, the *I*_K1_, *I*_NaSheP_ (Fig. 4e,f) and AP characteristics (Fig. 4g–i) of E-HDFs remained unchanged, demonstrating the stability of the acquired functional phenotype. The ability of these cells to undergo repeated freeze–thaw cycles without phenotypic alterations suggested the potential for off-the-shelf delivery applications. Electrophysiological phenotype of E-HDFs remained stable until the cells reached senescence (after additional eight passages and ∼10^6^-fold expansion; Supplementary Fig. 7a–d). Furthermore, using a classical heterologous expression system—monoclonal HEK293 line expressing Cx43, K_ir_2.1 and Na_v_SheP D60A—stable electrical properties were maintained beyond 20 passages (Supplementary Fig. 7e–g), with cells adapting to higher pacing rates by decreasing APD_80_ and CV (Supplementary Fig. 7h,i).

### Applicability to other sources of human unexcitable cells

To further demonstrate the versatility of this approach, we used the Kir2.1-Na_v_SheP D60N lentivirus to stably convert human ventricular fibroblasts (HVFs), human astrocytes (HAs) and HEK293s into engineered electrically excitable cells (E-HVFs, E-HAs and E-HEK293, respectively). All engineered cell types displayed strong *I*_NaSheP_ and *I*_K1_, and fired APs on stimulation (Supplementary Fig. 8). Expression of the Kir2.1 channels hyperpolarized the resting membrane potentials in all transduced cells to a similar value (Fig. 4j), whereas similar *I*_NaSheP_ levels resulted in comparable upstroke velocities (Fig. 4k). Significant differences in AP duration among different cell types (Fig. 4l) could be attributed to differences in their endogenous currents (Fig. 4m) and potentially other differences in epigenetic, posttranscriptional and posttranslational regulation of ion channel expression and function. Collectively, these results confirmed the robustness of the BacNa_v_ engineering approach against the starting unexcitable cell phenotype.

### E-Fibs improve conduction in model of interstitial fibrosis

**Figure 5:**
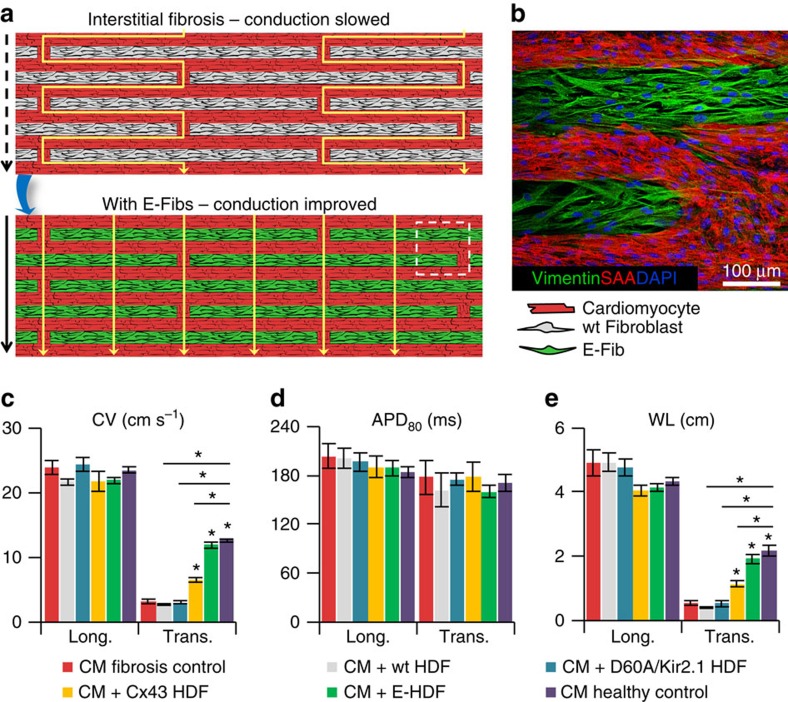
Improvement of AP conduction by E-Fibs in a model of cardiac interstitial fibrosis. (**a**) Schematic depicting use of E-Fibs to improve impaired conduction in an *in vitro* model of cardiac interstitial fibrosis. (**b**) Immunostaining corresponding to dashed-line square area in **a** showing vimentin^+^ E-HDFs in between sarcomeric α-actinin (SAA)^+^ neonatal rat cardiomyocytes (CMs). (**c**–**e**) Average CV (**c**), APD_80_ (**d**) and impulse wavelength (WL=CVxAPD_80_, **e**) values during longitudinal or transverse conduction in CM-only cultures (fibrosis control), CM+wt-HDF co-cultures, CM+Na_v_SheP D60A/Kir2.1-expressing HDF co-cultures, CM+Cx43-expressing HDF co-cultures, CM+E-HDF co-cultures and confluent anisotropic CM cultures (healthy control) (*n*=5–8). *P<0.001 versus transverse CVs in fibrosis control group. Error bars indicate s.e.m; statistical significance was determined by one-way analysis of variance, followed by Tukey's *post-hoc* test to calculate *P*-values.

To test whether E-Fibs could improve impaired AP conduction in native excitable tissues, we designed a zig-zag micropattern of neonatal rat cardiomyocytes (Fig. 5a and Supplementary Fig. 9a), which mimics the tortuous electrical conduction of cardiac tissue with interstitial fibrosis^24^,^25^. The cardiomyocyte zig-zag micropattern displayed normal AP propagation in the longitudinal direction (Supplementary Fig. 9b), but significantly slowed down transverse CV (3.2±0.4 cm s^−1^; Supplementary Fig. 9c), which was fully rescued (12.0±0.5 cm s^−1^) by the electrical coupling of actively conducting E-HDFs and cardiomyocytes (Fig. 5b,c, Supplementary Fig. 9g and Supplementary video 3). In contrast, unexcitable wt-HDFs or excitable Na_v_SheP D60A+Kir2.1 HDFs coupled only by endogenous junctions (no Cx43 overexpression) did not alter the transverse conduction (Fig. 5c and Supplementary Fig. 9d,e), whereas unexcitable HDFs with overexpressed Cx43 yielded only a small conduction improvement (to 6.5±0.3 cm s^−1^; Fig. 5c and Supplementary Fig. 9f). All groups had similar AP durations (Fig. 5d); thus, wavelength differences (Fig. 5e) reflected the differences in CV. Importantly, these studies suggested that slow electrical propagation in cardiac tissues with interstitial fibrosis could be significantly improved if fibroblasts were engineered to actively conduct APs by becoming both excitable and robustly coupled.

### BacNa_v_ augment and rescue mammalian tissue excitability

**Figure 6:**
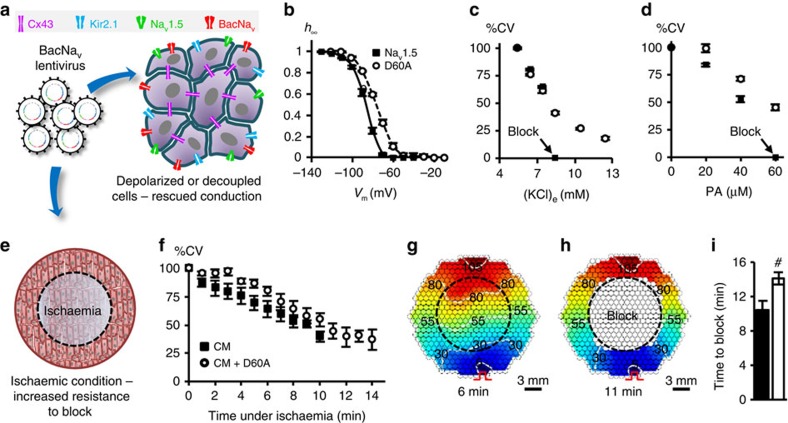
Improvement of mammalian AP conduction by BacNa_v_ in modeled pathological conditions. (**a**) Schematic depicting direct expression of BacNa_v_ in mammalian excitable tissues containing endogenous Na_v_1.5, to enhance conduction and prevent conduction block in simulated pathological conditions. (**b**) Steady-state inactivation (*h*_∞_) of Na_v_SheP D60A is more depolarized compared with that of Na_v_1.5 (*n*=5). (**c**,**d**) Conduction failure due to inactivation of Na_v_1.5 by membrane depolarization with elevated extracellular K^+^ (**c**, *n*=6) or blocking of intercellular coupling by PA (**d**, *n*=12–18) in Ex293 monolayers (black squares) is rescued by co-expression of Na_v_SheP D60A (white circles). (**e**) Schematic depicting exogenous expression of BacNa_v_ in neonatal rat CMs to increase resistance to conduction block in ischaemic conditions. Black dashed circle denotes position of glass coverslip used to induce regional ischaemia in CM monolayer. (**f**) Progressive conduction slowing until block with time of ischaemia in control (CM) and Na_v_SheP D60A transduced (CM+D60A) monolayers (*n*=6). (**g**,**h**) Representative isochrones maps of a CM monolayer showing conduction slowing after 6 min (**g**) and complete block after 11 min of ischaemia (**h**). Pulse signs indicate location of stimulating electrode. Circles denote 504 recording sites. (**i**) Under ischaemic condition, CMs transduced with Na_v_SheP D60A lentivirus (white) resisted conduction block longer than control CMs (black) (*n*=6, #*P*<0.01). Error bars indicate s.e.m; statistical significance was determined by an unpaired Student's *t*-test to calculate *P*-value.

Our computational results showed that select BacNa_v_ mutant (Na_v_SheP D60A) displayed comparable AP upstroke and slightly lower CV than mammalian Na_v_1.5 channel in an excitable cell model (Fig. 2d,e), suggesting that in addition to custom design of *de novo* excitable cells and tissues, BacNa_v_ channels could be exploited to directly manipulate or augment^26^,^27^ mammalian tissue excitability. Specifically, when lentivirally expressed in cardiomyocyte cultures, Na_v_SheP D60A channels significantly improved CV from 15.9±0.8 to 26.1±0.7 cm s^−1^ without altering AP duration (Supplementary Fig. 10). Furthermore, expression of BacNa_v_ channels could ameliorate reduced cardiac tissue excitability and arrhythmogenic conduction slowing in various pathological conditions (for example, ischaemia, infarction and congenital disease) associated with membrane depolarization and inactivation of Na_v_1.5 channels^2^,^28^,^29^ or diminished intercellular coupling^30^,^31^. For proof-of-concept studies, we used a Na_v_SheP D60A mutant, which exhibits ∼10 mV more depolarized inactivation *V*_1/2_ (−76.8 mV) compared with Na_v_1.5 channel (−87.4 mV) (Fig. 6a,b). Elevating extracellular K^+^ concentration to depolarize resting potential and inactivate Nav1.5 channels in Ex293 cells (a monoclonal HEK293 line engineered to stably express Kir2.1, Cx43 and Na_v_1.5 (ref. 32) resulted in conduction failure after addition of as little as 3 mM KCl. In contrast, Ex293 monolayers transduced with the Na_v_SheP D60A lentivirus sustained AP conduction at significantly higher extracellular K^+^ concentrations (up to 12.4 mM; Fig. 6c). Furthermore, Na_v_SheP D60A expression in Ex293 monolayers subjected to increased doses of gap junctional blocker palmitoleic acid (PA) attenuated conduction slowing and prevented conduction block at 60 μM PA when cells expressing only Na_v_1.5 channel failed to conduct (Fig. 6d). Finally, we tested whether viral expression of Na_v_SheP D60A could increase the resistance to conduction failure in cardiomyocyte cultures that underwent regional ischaemia after being covered with a glass coverslip^33^ (Fig. 6e). During ischaemia, cardiomyocytes exhibited gradual conduction slowing (Fig. 6f,g) and APD shortening (Supplementary Fig. 11a,b), followed by the partial (Supplementary Fig. 11c) and eventually complete conduction block (Fig. 6h and Supplementary Video 4). Importantly, Na_v_SheP D60A expression preserved cell excitability to significantly prolong the time to conduction block (from 10.5±1.0 min in control to 14.2±0.7 min in BacNa_v_-expressing cardiomyocytes; Fig. 6i).

## Discussion

Since their discovery 15 years ago^13^, prokaryotic Na^+^ channels have contributed greatly to understanding structure^16^,^20^, function^34^,^35^ and pharmacology^36^ of the more complex eukaryotic VGSCs. Yet, it has remained unexplored if large diversity and small gene size of BacNa_v_ could be employed to stably modify and augment the excitability of mammalian tissues, a feat difficult to achieve using large mammalian VGSCs. In this study, we presented a combined experimental and computation strategy for use of BacNa_v_ channels in the *de novo* induction and control of mammalian tissue excitability. Our goal was to develop a versatile platform that would be cost-effective, applicable to a variety of cell types and amenable to rapid production of large quantities of excitable cells for off-the-shelf use.

Previously, we applied a non-viral stepwise expression of cardiac Na^+^ channel Na_v_1.5, inward rectifier K^+^ channel Kir2.1 and gap junctional protein Cx43, combined with antibiotic selection, to generate a stable monoclonal HEK293 line capable of active AP conduction^32^. Other proof-of-concept studies applied adenoviral expressions of mammalian VGSCs to induce or modify excitability in primary tissues^37^,^38^,^39^; however, these effects were transient and unsuitable for long-term studies or the development of antiarrhythmic gene therapies^38^,^39^. Although the use of split-intein-mediated protein *trans*-splicing strategy^40^ might be adapted in the future to induce stable expression of mammalian VGSCs in primary cells, efficacy of this approach may be limited due to the need for co-transduction of two or more viruses to reconstitute functional channels. To circumvent the need for expressing large mammalian VGSCs, we exploited much smaller *BacNa*_*v*_ genes and designed high-titre multi-cistronic lentiviruses coding minimum sets of ion channels required for stable and efficient induction of excitability in primary cells. In fact, using BacNa_v_+Kir2.1 lentiviruses, human and rodent fibroblasts were routinely transduced with 90% efficiency without any loss of cell viability (not shown). Including a fluorescent reporter in the same virus allowed us to further purify transduced cells and fine-tune their electrical properties (Fig. 3 and Supplementary Fig. 5).

Importantly, lentivirally expressed ion currents in all engineered fibroblasts were much larger than their endogenous currents (Fig. 1). As such, resulting electrophysiological phenotype of engineered cells was relatively insensitive to the cell's epigenetic state and both predictable *in silico* and for the most part reproducible among different cell types *in vitro* (Fig. 4j–l and Supplementary Fig. 8). The ability to generate actively conducting HVFs, in particular, suggested a potential therapeutic strategy whereby *in situ* fibroblast engineering could improve impaired conduction in fibrotic heart tissues. As a proof of concept, we showed that well-coupled and excitable (but not weakly coupled and/or unexcitable) fibroblasts successfully restored normal neonatal rat cardiomyocyte conduction in an *in vitro* model of interstitial fibrosis (Fig. 5). On the other hand, distinct forms of cardiac fibroses, differing in size, and cellular and non-cellular components can result from various types of heart disease or injury^41^. *In situ* engineering of actively conducting fibroblasts, while potentially beneficial in improving cardiac conduction in small regions of interstitial or diffuse fibrosis where these cells come in close contacts with cardiomyocytes, may not prove as effective in large cardiac scars with low cellularity. Of note is also that without the prominent Ca^2+^ currents, engineered fibroblasts are unlikely to spontaneously fire APs or be a source of triggered activity, which could potentially make them less arrhythmogenic compared with the use of pluripotent stem cell-derived cardiomyocytes^42^,^43^.

Furthermore, transduction of mammalian excitable tissue cultures with specific BacNa_v_ mutants augmented or rescued conduction in healthy (Supplementary Fig. 10) and pathological (depolarization, decoupling and ischaemia; Fig. 6 and Supplementary Fig. 11) conditions, suggesting that BacNa_v_-based gene therapies could be targeted directly to excitable cells. In addition, previous studies have shown that BacNa_v_ could be converted into Ca^2+^-selective channel (BacCa_v_) by substituting three amino acid residues of the selectivity filter with Aspartate^19^,^44^,^45^. Hence, stable expression of engineered BacNa_v_ channels or their Ca^2+^-selective derivatives may enable direct therapeutic effects on excitable and unexcitable cells whose function relies on voltage-gated Na^+^ or Ca^2+^ channels.

Although in this study we focused on altering specific amino acid residues (E43 or D60) in a particular BacNa_v_ orthologue (Na_v_SheP), various combinations of other BacNa_v_ orthologues and residues^18^,^46^,^47^ could be explored to customize phenotype of engineered or native excitable cells. For example, mutations in the ‘glycine hinge' region in some BacNa_v_ orthologues could change channel inactivation rate^15^, and along with tuning of Kir2.1 expression via an independent promoter, could be used to control AP duration to avoid arrhythmogenic dispersion of refractoriness if E-Fibs were used for cardiac cell or *in situ* gene therapy. Furthermore, replacing the highly conserved negative residues (E70 and D91) of BacNa_v_ voltage-sensitive domain with positively charged residues could shift activation to more hyperpolarized voltages^48^ and be used to reduce AP threshold. Similarly, substituting the negatively charged residues in the neck region of the carboxy-terminal domain (CTD) of the BacNa_v_ channel into glycine could destabilize the helix conformation of this region, decrease the depolarization energy required for channel opening^49^ and further augment cell excitability and conduction. Furthermore, a recent study by Arrigoni *et al*.^50^ showed that BacNa_v_ CTD plays a critical role in voltage dependence of activation, suggesting CTD swapping among different orthologues could provide yet another method to control excitability of engineered cells.

Basic studies in ‘lower' organisms, including prokaryotes, have led to versatile approaches to manipulate and study mammalian biology. Recent examples include optogenetics^51^ and genome-editing techniques^52^ that, in addition to becoming powerful research tools, have been extensively investigated for potential medical applications. In line with these undertakings, our work supports expansion of prokaryotic ion channel research to basic studies of mammalian tissue excitability and development of new human therapies.

## Methods

### Screening of bacterial sodium channels

To engineer excitable cells, we first explored use of NaChBac channel, the longest studied BacNa_v_ orthologue^13^,^17^,^45^,^53^. However, its slow recovery from inactivation yielded excitable cells with very low maximum capture rate (<1 Hz). This prompted us to instead use Na_v_RosD G217A and Na_v_SheP channels that were kindly provided by Dr Katsumasa Irie from Kyoto University^15^. Libraries of SheP(D60X) and SheP(E43X) mutants were generated using Quikchange II XL Site-Directed Mutagenesis Kit (Agilent Technologies), as per the manufacturer's instructions. All the primers used are listed in Supplementary Table 2. Each gene was subcloned into the mammalian expression vector pCMV5(CuO)-IRES-GFP (Qbiogene) and transfected into HEK293 cells stably expressing Kir2.1 using Lipofectamine 2000 transfection reagent (Life Technologies). GFP fluorescence was usually detected after 12 h and cells were patch clamped 24 h after transfection, to derive AP parameters.

### Lentivirus production

Lentiviral plasmids were constructed from the pRRL-CMV vector (a gift from Dr Inder Verma, Salk Institute). BacNa_v_ (Na_v_RosD G217A or Na_v_SheP mutant, without a stop codon) was ligated with eGFP-Kir2.1 (Kir2.1 fused with eGFP at the amino terminus) via the viral P2A peptide^54^ before being subcloned into pRRL-CMV, to create the bi-cistronic lentiviral plasmid pRRL-CMV-BacNa_v_-P2A-eGFP-Kir2.1. To enhance gap junction expression in engineered cells, Cx43 (ref. 32) was linked with mCherry via P2A and subcloned into the pRRL-CMV lentiviral plasmid (pRRL-CMV-Cx43-P2A-mCherry). In the main text, BacNa_v_-Kir2.1 and Cx43 lentiviruses refer to pRRL-CMV-BacNa_v_-P2A-eGFP-Kir2.1 and pRRL-CMV-Cx43-P2A-mCherry virus, respectively. Other lentiviral constructs used in the study were pRRL-CMV-BacNa_v_-P2A-Kir2.1-T2A-eGFP and pRRL-CMV-BacNa_v_-T2A-eGFP. High-titre lentiviruses were produced using second generation lentiviral packaging system. Briefly, 293FT cells (Life Technologies, R700-07) were co-transfected with lentiviral plasmid, packaging plasmid psPAX2 and envelope plasmid pMD2.G (2:1:1 mass ratios) using Lipofectamine 2000 (Life Technologies). Supernatant containing lentiviral particles was collected 72 h after transfection, centrifuged (500 *g*, 10 min) and filtered through 0.45 μm cellulose acetate filter (Corning) to remove cell debris before combined with Lenti-X Concentrator (Clontech) at 3:1 volume ratio for overnight incubation at 4 °C. Concentrated lentiviral particles were harvested following 45 min centrifugation (1,500 *g*, 4 °C) and resuspended in 1/10 to 1/100 of the original volume in DMEM medium. Plasmids psPAX2 and pMD2.G were obtained from Didier Trono (Addgene plasmids #12260 and #12259).

### Generation of engineered human fibroblasts

Primary adult HDFs (Gibco, C-013-5C) were co-transduced with BacNa_v_-Kir2.1 and Cx43 lentiviruses at a density of 10^4^ cells per cm^2^ in fibroblast basal medium (ATCC, PCS-201-030). Five days after lentiviral transduction, GFP^+^/mCherry^+^ cells were sorted by FACS analysis, replated in fibroblast growth medium (basal medium supplemented with fibroblast low-serum growth kit, ATCC, PCS-201-041) and assessed with patch-clamp recordings and optical mapping at 1 week and 4 weeks after sorting. Conversion of HDFs into myofibroblasts was induced by adding transforming growth factor-β (10 ng m^l−1^) into culture media for 5 days. Similar lentiviral transduction protocol was repeated in HVFs (Lonza, CC-2904), HAs (Lonza, CC-2565) and HEK293 cells (ATCC, CRL-1573).

### Isotropic and anisotropic monolayers of HDFs

E-HDFs were seeded onto 22 mm-diameter Aclar (Electron Microscopy Sciences) coverslips coated with fibronectin (Sigma, 15 μg ml^−1^, 30 min) at a density of 5 × 10^4^ cells per cm^2^ to form isotropic monolayers. Anisotropic E-HDF monolayers were formed on Aclar coverslips microcontact printed with a pattern of parallel fibronectin lines (10 μm width, 10 μm spacing) as previously described^55^. Cells were cultured for 24 h to become confluent and form intercellular junctions followed by optical mapping of AP propagation and immunostaining.

### Micropatterned zig-zag cultures

All animals were treated in accordance with protocols approved by the Duke University Institutional Animal Care and Use Committee. Neonatal rat cardiomyocytes were enzymatically dissociated from ventricles of 2-day-old Sprague–Dawley rats and enriched using differential pre-plating^56^. Specifically, ventricles were excised, minced and incubated with 0.1% trypsin overnight and dissociated in four sequential steps using 0.1% collagenase. Isolated cells were centrifuged (5 min, 200 *g*) and further enriched by two 45 min pre-plating steps to select for cardiomyocyte-enriched cells that remained unattached after the second pre-plating. Zig-zag cultures were generated as previously described^25^. Briefly, Aclar coverslips (22 mm diameter) were coated with 5 μg ml^−1^ collagen I (rat tail, BD Biosciences) for 30 min, rinsed with PBS, then micro-contact printed with a zig-zag network of fibronectin (100 μm-wide lines spaced 100 μm apart and connected by 100 μm-wide transverse bridges staggered every 6 mm). Cardiomyocytes were seeded at a density of 2 × 10^5^ cells per cm^2^ in DMEM/F-12 medium (Gibco, 11320-033) supplemented with 10% calf serum and 10% horse serum, followed by a switch to 5% fetal bovine serum media at culture day 2. The following day, E-HDFs were added to the cardiomyocyte zig-zag cultures at a density of 2.5 × 10^4^ cells per cm^2^. On day 5, co-cultured monolayers were assessed for AP propagation by optical mapping and subsequently fixed for immunostaining.

### Immunostaining

Cell monolayers or tissue patches were fixed in 2% paraformaldehyde (15 min), permeabilized in 0.5% Triton-X (30 min) and blocked in a 5:1 solution of 1% BSA and chicken serum (30 min). The following primary antibodies (1 h incubation) were used: anti-sarcomeric α-actinin (Sigma, a7811, 1:200), anti-Cx43 (Life Technologies, 71-0700, 1:100), anti-vimentin (Sigma, v6630, 1:500), anti-smooth muscle actin (Sigma, a2547, 1:200), anti-GFAP (BD Biosciences, 561483, 1:100) and anti-Ki67 (Abcam, ab15580, 1:200). Secondary antibodies (1 h incubation) included the following: Alexa488 (Life Technologies, A-21200 or A-21441, 1:200), Alexa594 (Life Technologies, A-21201 or A-21442, 1:200), Alexa647 (Life Technologies, A-21463, 1:200), Alexa488-conjugated phalloidin (Life Technologies, A12379, 1:300) and 4,6-diamidino-2-phenylindole (Sigma, 1:300). All immunostaining steps were performed at room temperature. Fluorescence images were acquired using inverted fluorescence (Nikon TE2000) or confocal (Leica SP5) microscope and processed with ImageJ software.

### Fluorescence recovery after photobleaching

Functional coupling in wt and Cx43-expressing HDFs was assessed using fluorescence recovery after photobleaching^21^,^57^ as previously described^58^. Briefly, confluent HDF monolayers were stained with Calcein AM dye (Molecular Probes, 0.5 μM in DMEM) for 20 min at 37 °C, washed with PBS and immersed again in fresh media. The target cell was photobleached with a 488 nm Argon laser and subsequently imaged every 15 s for 6 min using an upright confocal microscope (Zeiss, LSM 510). The level of fluorescence in the target cell was normalized to the fluorescence level in remote unbleached cells and plot as a function of time post photobleaching.

### Whole-cell patch-clamp recordings

Cells were briefly trypsinized, plated onto poly-L-lysine-coated glass coverslips and left to attach for 5 h. Cells were then transferred to a patch-clamp chamber perfused with Tyrode's solution containing (in mM): 135 NaCl, 5.4 KCl, 1.8 CaCl_2_, 1 MgCl_2_, 0.33 NaH_2_PO_4_, 5 HEPES and 5 glucose. Patch pipettes were fabricated with tip resistances of 1–2 MΩ when filled with pipette solution consisting of (in mM): 140 KCl, 10 NaCl, 1 CaCl_2_, 2 MgCl_2_, 10 EGTA, 10 HEPES and 5 MgATP. Whole-cell voltage-clamp and current-clamp recordings were acquired at room temperature (25 °C) and 37 °C, respectively, using the Multiclamp 700B amplifier (Axon Instruments), filtered with a 10-kHz Bessel filter, digitized at 40 kHz and analysed using WinWCP software (John Dempster, University of Strathclyde). To measure BacNa_v_ activation properties, membrane voltage was stepped from a holding potential of −100 mV to varying 500 ms test potentials (−50 to 60 mV, increments of 10 mV). Inactivation of BacNa_v_ was derived from peak currents measured at 0 mV after varying 3 s prepulse potentials (−160 to −30 mV, increments of 10 mV). For the channels with more hyperpolarized activation and inactivation (Na_v_SheP WT and D60E), holding voltage for steady-state activation protocol was set at −160 mV, whereas steady-state inactivation protocol included prepulse potentials from −180 mV to −30 mV. Steady-state *I*_K1_–*V* curve was constructed from the current responses to varying 1 s test potentials (−130 to 50 mV, increments of 10 mV) from a holding potential of −40 mV. APs were elicited by injecting a 1 ms current pulse at 1.1 × threshold amplitude.

### Optical mapping of AP propagation

Cell monolayers or zig-zag cultures were optically mapped with a 20 mm diameter hexagonal array of 504 optical fibres (Redshirt Imaging), as previously described^32^,^55^,^56^. Specifically, cultures were stained with a voltage-sensitive dye (Di-4-ANEPPS, 10 μM) for 5 min at room temperature, transferred to a recording chamber filled with Tyrode's solution at 37 °C and illuminated by a solid-state excitation light source (Lumencor, SOLA SM) filtered through a 520±30 nm bandpass filter. Emitted red fluorescence signals (*λ*>590 nm) were collected by the optical fiber array, converted to voltage signals by photodiodes and recorded at a 2.4 kHz sampling rate with a 750 μm spatial resolution. AP propagation was triggered by a 10 ms, 1.2 × threshold, 1 Hz stimulus from either a bipolar point electrode (in isotropic or anisotropic monolayers of E-HDFs) or a line electrode (in the zig-zag cultures). Generation of isochrone maps and calculation of CV, AP duration at 80% repolarization (APD_80_) and impulse wavelength were performed using custom MATLAB software, as previously described^59^,^60^.

### Depolarization and decoupling studies

Similar to previous studies^61^, membrane depolarization in Ex293 monolayers was induced by applying increasing doses of KCl (in 1 mM increments starting from 5.4 mM). After 5 min equilibration at each KCl dose, cells were paced at 1 Hz and AP conduction optically mapped. For gap junction decoupling studies, PA (Sigma, 76168) was dissolved in dimethyl sulfoxide and sonicated (10 min, 37 °C) to yield a stock concentration of 50 mM. Ex293 monolayers were exposed to increasing doses of PA (0, 20, 40 and 60 μM) and after 15 min equilibration at each dose, cells were paced at 1 Hz and AP conduction optically mapped.

### Regional ischaemia studies

Regional ischaemia in neonatal rat cardiomyocyte monolayers was induced as described previously^33^,^62^. Briefly, 12 mm round glass coverslip was placed onto the central area of a 22 mm cardiac monolayer to induce regional ischaemia. Cells were continuously paced at 1 Hz and AP conduction optically mapped every 1 min during ischaemia, until complete conduction failure in the ischaemic region. CV, APD_80_ and optical AP amplitude measured at the recording sites of the central ischemic region were normalized to corresponding baseline values before ischemia. Recording site was defined as inactive (not conducting) if its AP amplitude failed below 10% of baseline value.

### Computational modelling

Experimentally derived modelling parameters are listed in Supplementary Table 3. The AP generation in HEK293 cells co-expressing K_ir_2.1 and BacNa_v_ channels was simulated using the Hodgkin–Huxley formalism^63^ and the following differential equation:


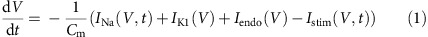


where *V* is the cell transmembrane potential, *C*_m_ is the total membrane capacitance, *I*_Na_ is the BacNa_v_ channel current, *I*_K1_ is the inward-rectifier potassium current, *I*_endo_ is the endogenous background current and *I*_stim_ is the stimulus current. Based on the similarities between BacNa_v_ and mammalian Ca_v_ channels^13^,^45^, the BacNa_v_ conductance was modelled as a simple product of activation and inactivation gating variables and *I*_Na_ was expressed using the following equation:


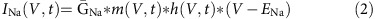


where 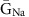 is the maximum conductance of the BacNa_v_ channel, *m* and *h* are the first-order activation and inactivation gating variables, respectively, and *E*_Na_ is the sodium Nernst potential given in Supplementary Table 3. Mathematical model of human Na_v_1.5 was adopted from a previous model of human ventricular tissue^64^ with a slightly modified inactivation time constant to better fit voltage clamp recordings in Na_v_1.5-expressing HEK293 cells.

From the voltage-clamp activation and inactivation protocols, the steady-state values of the gating variables as a function of V were plotted and fit to the Boltzmann equation^63^, to derive the midpoint voltage and slope factor listed in Supplementary Table 1:


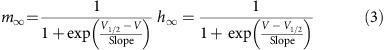


The initial and late phases of *I*_Na_ traces for each clamp potential were fit as single exponentials to derive time constants of activation (*τ*_m_) and inactivation (*τ*_h_) as functions of *V*. The *τ*_m_ and *τ*_h_ at *V*=+20 mV are listed in Supplementary Table 1.

Voltage-clamp recordings of inward-rectifying potassium current were used to fit steady-state *I*_K1_ values to a time-independent Nygren *et al*.^65^ model as follows:


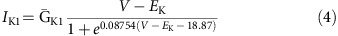


where 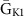 is the maximum conductance of the K_ir_2.1 channel and *E*_K_ is the potassium Nernst potential given in Supplementary Table 3.

Finally, the endogenous background current of wt HEK293 or HDF cells, *I*_endo_ (in pA), was fit by a time-independent linear equation:


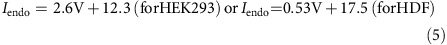


where *V* is given in millivolts. Simulations of single-cell APs for different BacNa_v_ mutants were performed using constant 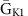 and 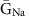 values (measured by patch clamp in a monoclonal HEK293 line expressing K_ir_2.1, Cx43 and Na_v_SheP D60A; Supplementary Table 3), to allow direct comparisons of how kinetic properties of BacNa_v_ on their own affect AP characteristics (upstroke velocity, APD_80_ and threshold potential). As the whole-cell voltage clamp recordings were performed at 25 °C, to model AP generation at 37 °C, we scaled up *E*_K_ and *E*_Na_ from Supplementary Table 3 by 310.15 °K/298.15 °K and further scaled *I*_Na_, *I*_K1_ and *I*_endo_ using *Q*-values of 2.7, 1.8 and 2.1, respectively.

To simulate AP propagation in HEK293 cells expressing different BacNa_v_ mutants, we applied a continuous cable equation discretized using forward Euler's method^66^,^67^,^68^ as follows:


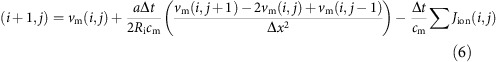


where Δ*t* and Δ*x* are the time (index *i*) and space (index *j*) discretization steps, respectively, *c*_m_ is the specific membrane capacitance, *a* is the cable radius, *R*_i_ is the intracellular resistivity, and 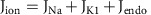 is the total ionic current density (in pA cm^−2^), calculated by dividing the total ionic current 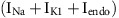 from equation (1) with total membrane surface area. The *R*_i_ value was adopted to simulate the mean CV measured in optically mapped monolayers of a monoclonal HEK293 line expressing K_ir_2.1, Cx43 and Na_v_SheP D60A. All simulations were performed with the identical model parameters given in Supplementary Table 3 and distinct kinetic properties measured in different BacNa_v_ mutants (adjusted to 37 °C as described above).

The total length of the simulated cable was 0.72 cm (that is, 300 nodes). The first three nodes in the cable were stimulated by current injection (1.2 × threshold) at 1 Hz rate and activation time at each node was determined as the time of maximum upstroke velocity of propagated AP. Inverse slope of activation time versus distance line fit in the central 0.24 cm of the cable was used to calculate CV.

### Statistical analysis

All data are presented as mean±s.e.m. and statistical significance was determined by one-way analysis of variance, followed by Tukey's *post-hoc* test to calculate *P*-values. Statistical significance was defined as ^^^*P*<0.05, ^#^*P*<0.01 and *P<0.0001.

### Data availability

All data supporting the findings of this study are available within the article and its Supplementary Information files or can be requested from the authors.

## Additional information

**How to cite this article**: Nguyen, H. X. *et al*. Engineering prokaryotic channels for control of mammalian tissue excitability. *Nat. Commun.* **7**, 13132 doi: 10.1038/ncomms13132 (2016).

## Supplementary Material

Supplementary Information Supplementary Figures 1-11 and Supplementary Tables 1-3.

Supplementary Movie 1 Action potential propagation in monolayers of E-HDFs expressing Kir2.1, Cx43, and Na_v_RosD G217A.

Supplementary Movie 2 Action potential propagation in monolayers of E-HDFs expressing Kir2.1, Cx43, and Na_v_SheP D60N.

Supplementary Movie 3 E-HDFs rescue impaired cardiomyocyte conduction *in vitro*.

Supplementary Movie 4 Regional ischemia in neonatal rat cardiomyocyte monolayer.
