# Supplementary material for: Engineering prokaryotic channels for control of mammalian tissue excitability
Source: Nat Commun. 2016 Oct 18;7:13132. doi: 10.1038/ncomms13132 (PMC5071848; doi:10.1038/ncomms13132)
Supplement: Supplementary Information — Supplementary Figures 1-11 and Supplementary Tables 1-3. [file ncomms13132-s1.pdf]

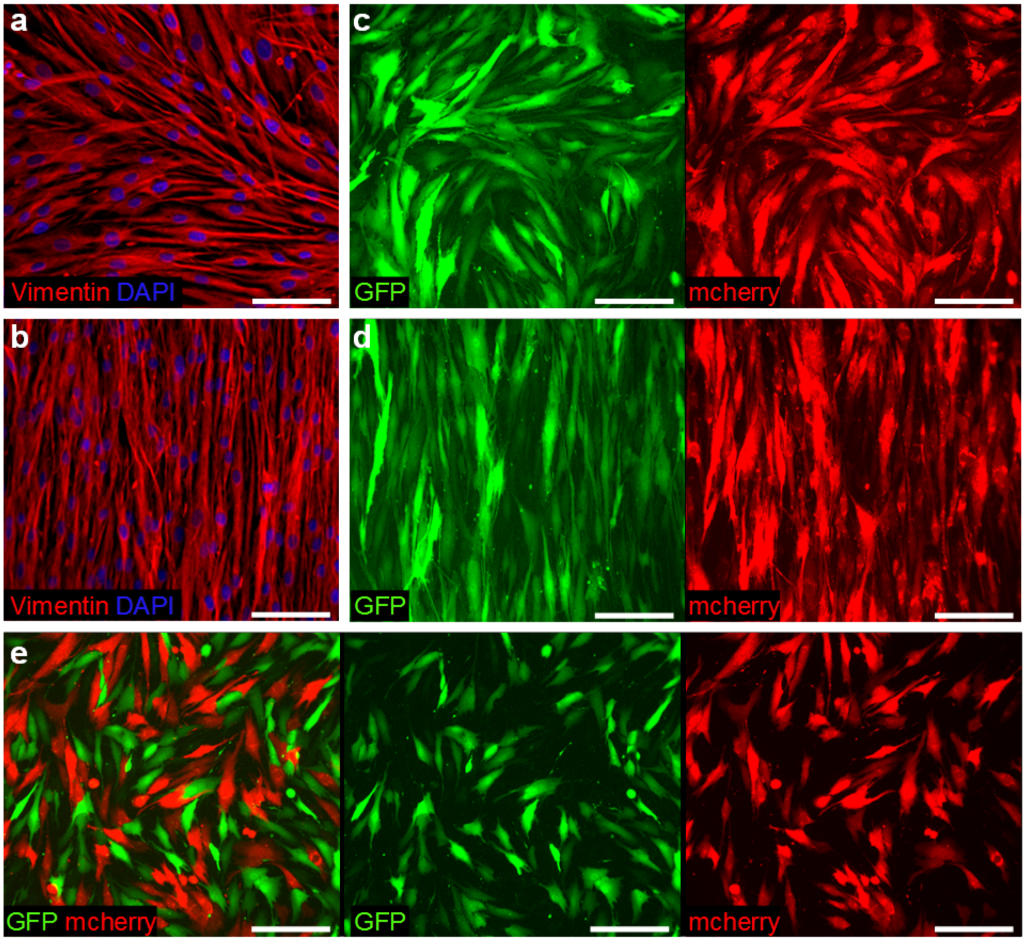

Supplementary Figure 1 | Structure and lentiviral co-transduction of E-HDF monolayers. (**a**,**b**) Representative images of Vimentin^{+} E-HDFs in isotropic (**a**) or anisotropic (**b**) monolayers. (**c**,**d**) Strong, ubiquitous co-expression of eGFP (left) and mCherry (right) in isotropic (**c**) and anisotropic (**d**) E-HDF monolayers co-transduced with pRRL-CMV-NavSheP D60N-P2A-Kir2.1-T2A-eGFP and pRRL-CMV-Cx43-P2A-mCherry lentiviruses. (**e**) Mixed culture of HDFs transduced with pRRL-CMV-NavSheP D60N-P2A-Kir2.1-T2A-eGFP and HDFs transduced with pRRL-CMV-Cx43-P2A-mCherry virus shown as a control to demonstrate no cross-talk between read and green channels. Scale bars, 100 μm.

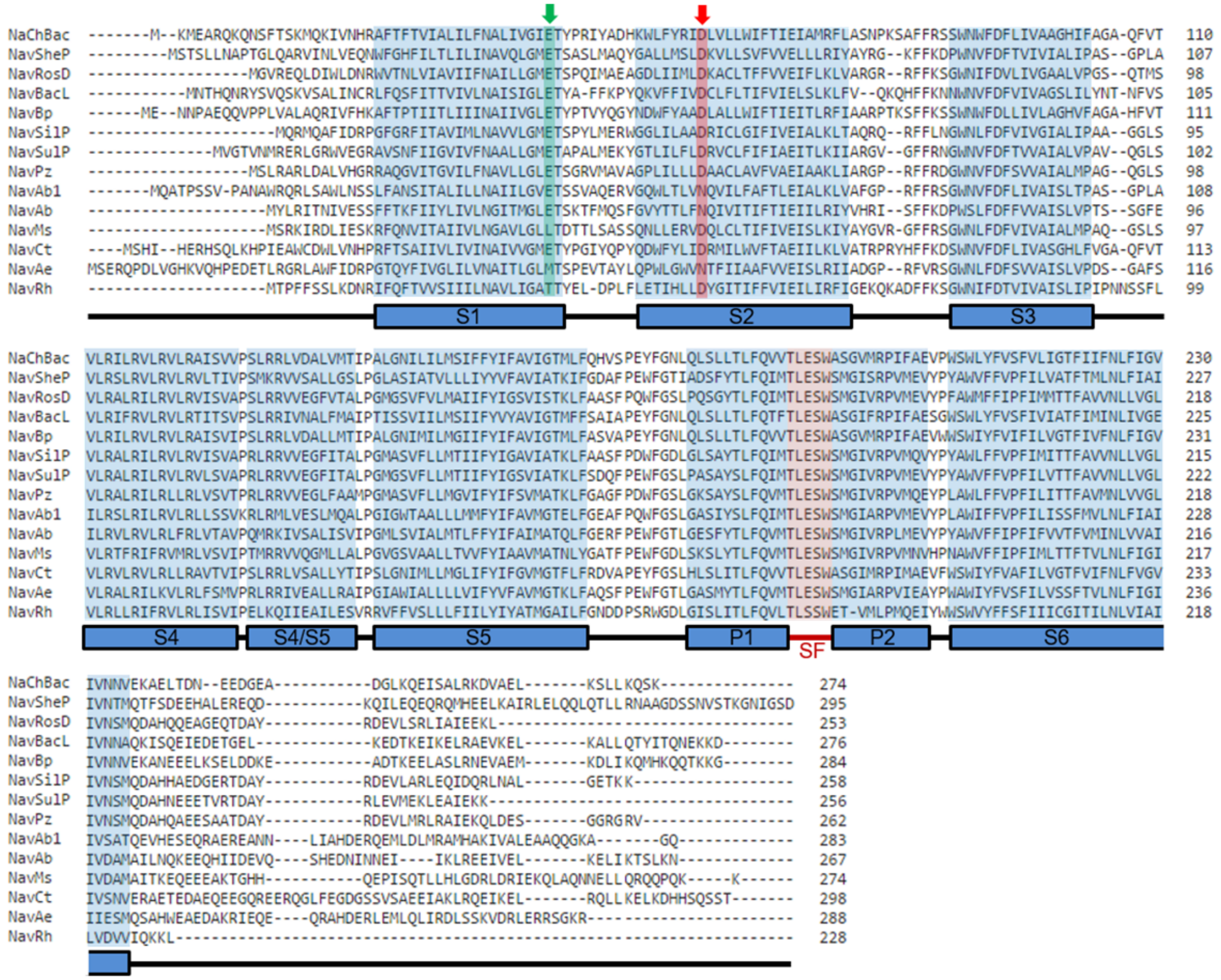

Supplementary Figure 2 | Homology of different BacNa$_v$ orthologs. Residues E43 and D60 are indicated by green and red arrows respectively. The main regions labeled are: S1-S6 transmembrane helices, S4/S5 linker, P1 and P2 pore helices, and selectivity filter (SF). The BacNa$_v$ orthologs listed have been previously characterized either structurally or functionally: NaChBac (*Bacillus halodurans*), Na$_v$SheP (*Shewanella putrefaciens*), Na$_v$RosD (*Roseobacter denitrificans*), Na$_v$BacL (*Bacillus licheniformis*), Na$_v$Bp (*Bacillus pseudofirmus*), Na$_v$SilP (*Silicibacter pomeroyi*), Na$_v$SulP (*Sulfitobacter pontiacus*), Na$_v$Pz (*Paracoccus zeaxanthinifaciens*), Na$_v$Ab1 (*Alcanivorax borkumensis*), Na$_v$Ab (*Arcobacter butzleri*), Na$_v$Ms (*Magnetococcus marinus*), Na$_v$Ct (*Caldalkalibacillus thermarum*), Na$_v$Ae (*Alkalilimnicola ehrlichii*), and Na$_v$Rh (*Rickettsiales sp.*)

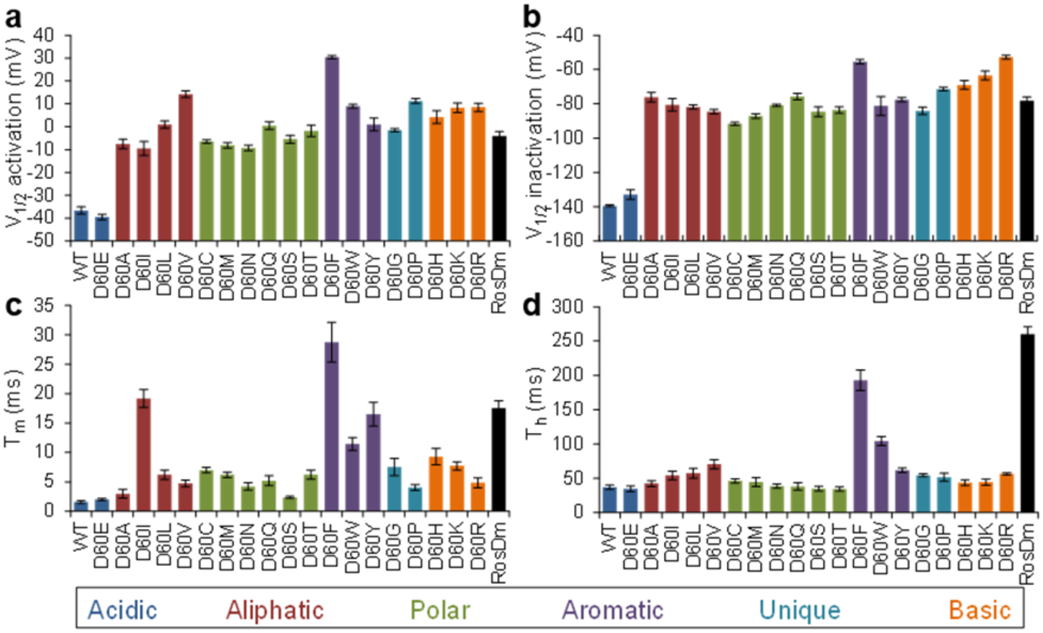

Supplementary Figure 3 | Electrophysiological properties of Na$_v$SheP mutants. (**a**,**b**) Mid-points ($V_{1/2}$) of activation (**a**) and inactivation (**b**) curves of Na$_v$SheP mutants exhibit different depolarization shifts relative to the $V_{1/2}$ of the wild-type (WT) channel. (**c**,**d**) Time constants of activation (τ_m, **c**) and inactivation (τ_h, **d**) measured at +20mV. Color-coding into groups is based on the characteristics of the respective amino acid side chains at residue 60. Recordings were performed at 25^{0}C (n = 4-10). Supplementary Table 1 shows a complete list of all activation and inactivation parameters. Error bars indicate s.e.m.

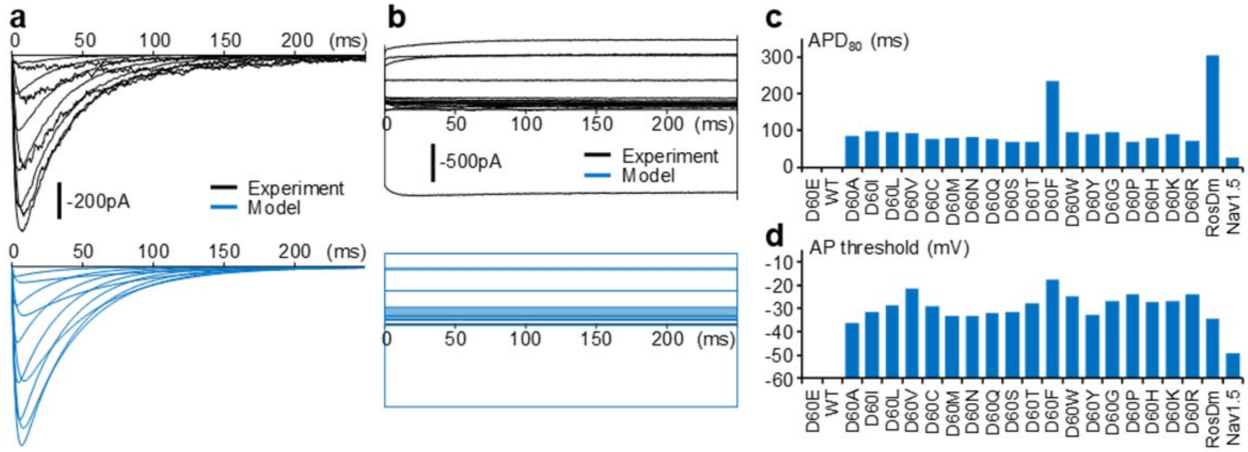

Supplementary Figure 4 | Computational modeling of E-HDF electrophysiology. (**a**) Representative whole-cell voltage clamp recording of Na_vSheP D60A current in E-HDFs and the corresponding computational traces. (**b**) Representative whole-cell voltage clamp recording of I_K current in E-HDFs and the corresponding computational traces. (**c**,**d**) AP characteristics of E-HDFs expressing different BacNa_v mutants or Na_v1.5, obtained by computational modeling: APD_{80} (**c**) and AP threshold (**d**). For modeling BacNa_v currents, parameters derived from voltage-clamp measurements at 25^0C were scaled for the temperature of 37^0C. Highly hyperpolarized inactivation of WT and D60E channels prevented AP initiation.

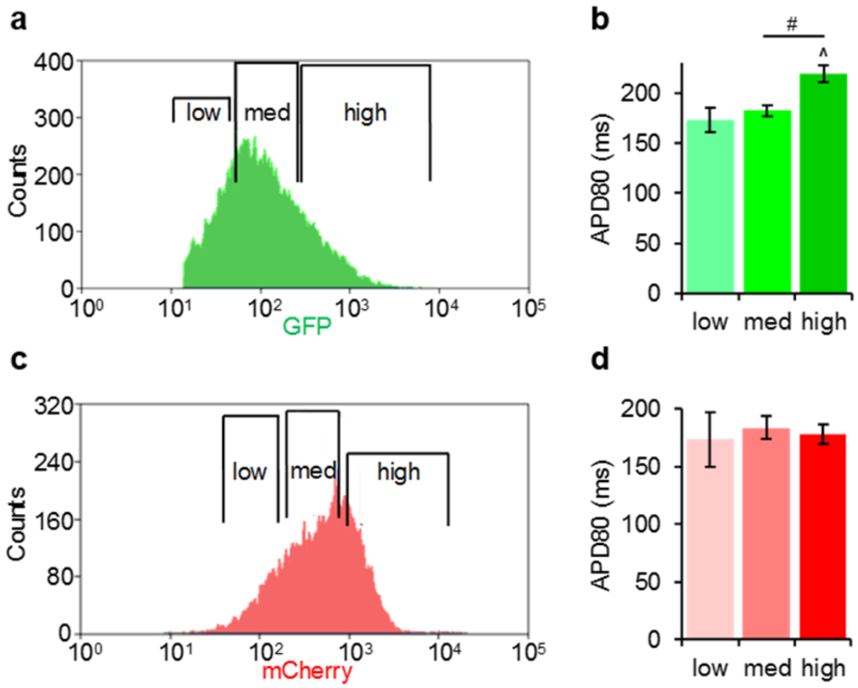

Supplementary Figure 5 | Use of FACS to tune electrophysiological properties of E-Fibs. (**a**) E-HDFs generated by co-transduction with Na_vSheP D60N-P2A-eGFP-Kir2.1 and Cx43-P2A-mCherry were sorted into three groups based on the level of eGFP expression (low, medium, high). (**b**) Optical mapping of eGFP-sorted E-HDFs showed longer APD_{80} associated with higher eGFP intensity (n = 6-10). (**c-d**) When E-HDFs were sorted into three groups based on mCherry expression level (low, medium, high, **c**), APD_{80} was comparable among the groups (**d**, n = 5). $^{\#}P<0.01$; $^{\wedge}P<0.05$ vs low eGFP group in **b**. Error bars indicate s.e.m; statistical significance was determined by one-way ANOVA, followed by Tukey's *post hoc* test to calculate P values.

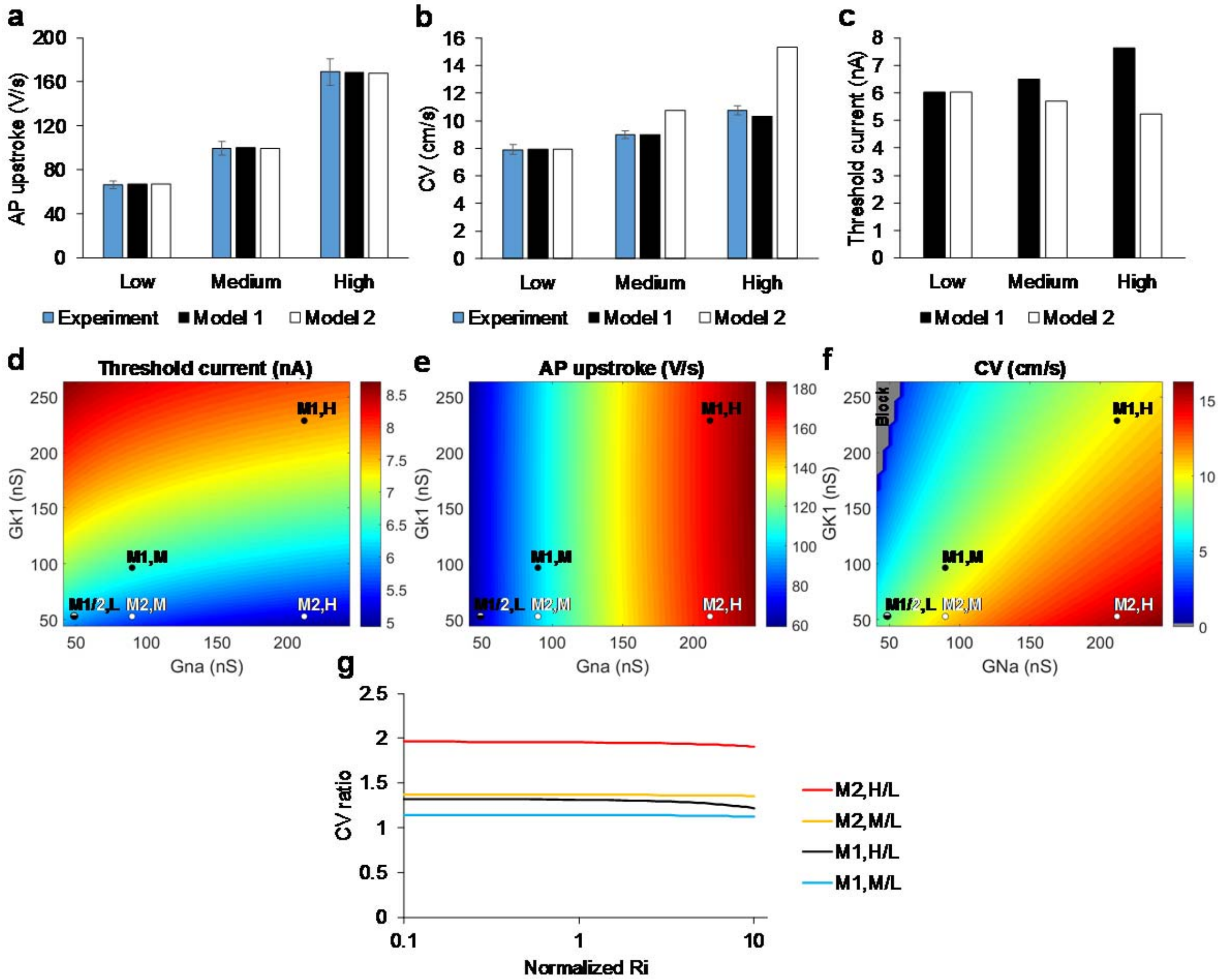

Supplementary Figure 6 | Computational studies of BacNa$_v$ and Kir2.1 effects on E-HDF electrical properties. (**a**-**c**) Maximum AP upstroke velocity (**a**), conduction velocity (**b**), and threshold current required to elicit AP (**c**) in E-HDFs expressing varying levels (low, medium, and high) of both Na$_v$SheP D60N and Kir2.1 (model 1) or the low level of Kir2.1 and varying levels of only Na$_v$SheP D60N (model 2). Experimental data for AP upstroke and CV shown in Fig. **3e,h** are included for comparison. (**d**-**f**) Simulated effects of varying BacNa$_v$ and Kir2.1 conductance values (G_{Na} and G_{K1}) on threshold current (**d**), AP upstroke (**e**), and CV (**f**). Solid circles denote low (L), medium (M), or high (H) groups using either model 1 (M1, black circles) or model 2 (M2, white circles) shown in panels **a**-**c**. (**g**) Effects of varying intercellular resistivity, R_i (shown relative to nominal R_i value used in panels **b** and **f**), on CV ratios between the groups. Error bars indicate s.e.m.

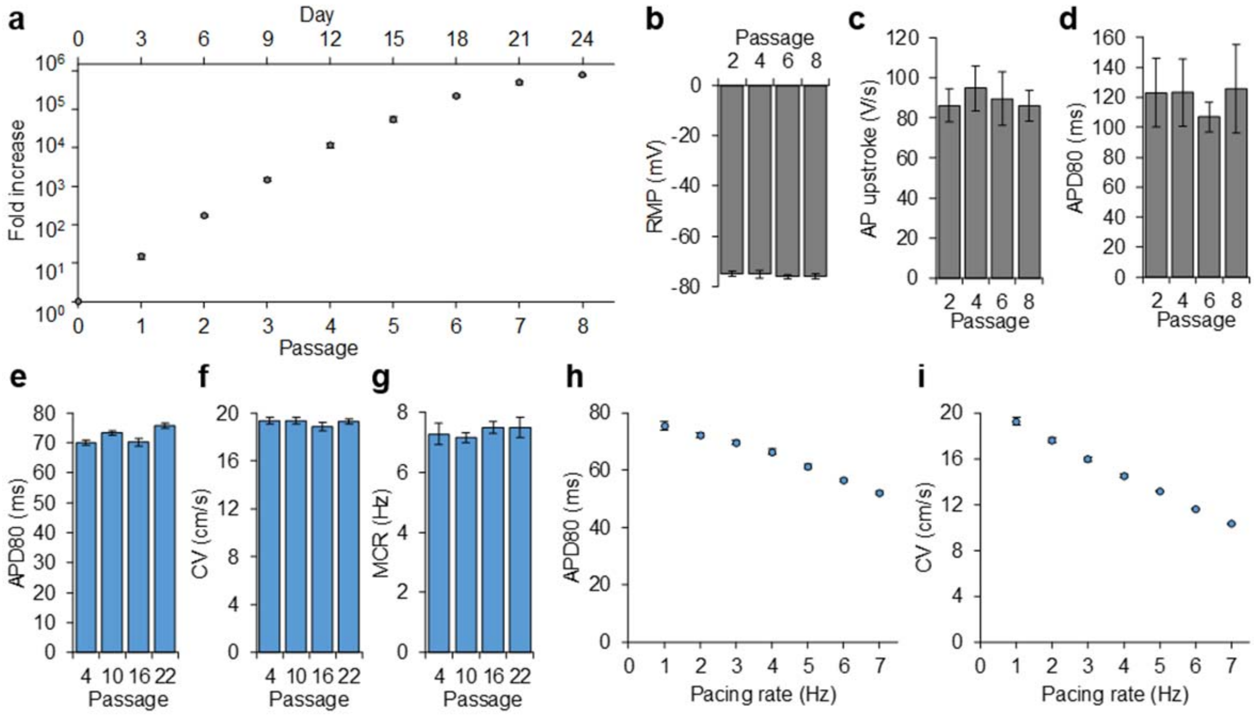

Supplementary Figure 7 | Stability of engineered cell phenotypes during long-term expansion. (**a**) Growth curve of E-HDFs transduced with Na$_v$SheP D60N-Kir2.1 lentivirus following initial expansion and FACS. Cells senesced at passage eight. (**b-d**) Resting membrane potential (**b**), maximum AP upstroke velocity (**c**), and APD$_{80}$ (**d**) of E-HDFs at various passages throughout expansion assessed by patch clamp recordings (n = 8-10). (**e-g**) Optical mapping studies in monolayers of monoclonal E-HEK293 cells expressing Na$_v$SheP D60A, Kir2.1, and Cx43, showing stable APD$_{80}$ (**e**), CV (**f**), and maximum capture rate (MCR, **g**) during 22 passages (n = 6-8). Properties beyond passage 22 were not examined and were presumed to remain stable. (**h**,**i**) Dependence of APD$_{80}$ (**h**) and CV (**i**) on pacing rate in monolayers made from passage 22 E-HEK293 cells (n = 6). AP conduction at each pacing rate was mapped after 30s of pacing. All data were obtained at 37^{0}C. Error bars indicate s.e.m; statistical significance was determined by one-way ANOVA.

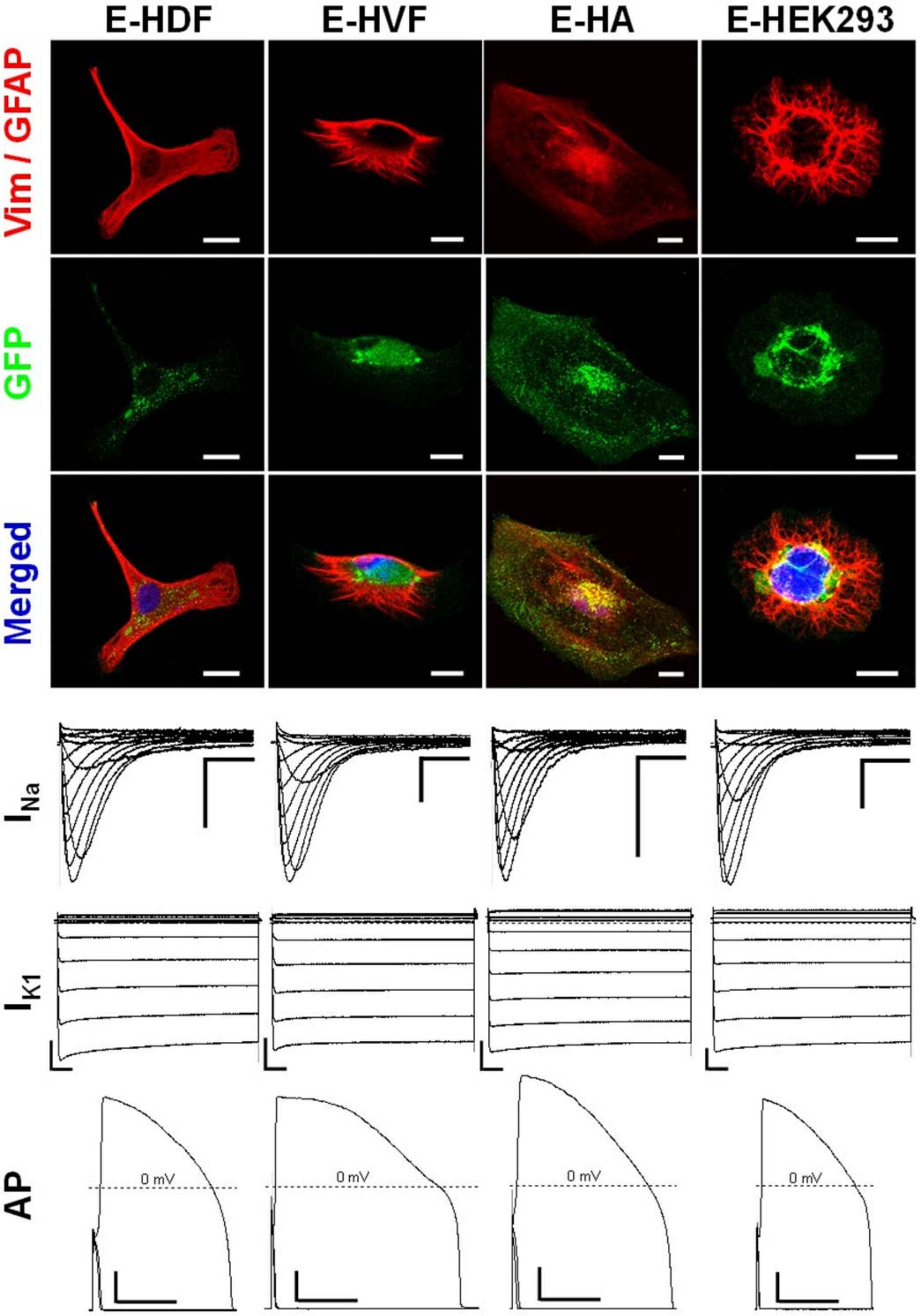

Supplementary Figure 8 | Induction of excitability in various types of unexcitable human cells. Each human cell type (shown in different columns) was transduced with Na_vSheP D60N-P2A-eGFP-K_{ir}2.1 lentivirus, expanded, and analyzed by voltage and current clamp recordings.

Engineered human astrocytes (E-HAs) were labeled for Glial fibrillary Acidic Protein (GFAP), while engineered human dermal fibroblasts (E-HDFs), human ventricular fibroblast (E-HVFs), and human embryonic kidney 293 cells (E-HEK293s) were labeled for vimentin. Scale bars: 20µm (immunostaining images), 100ms and 2 nA (I_{Na} and I_K recordings), 25ms and 20mV (AP recordings).

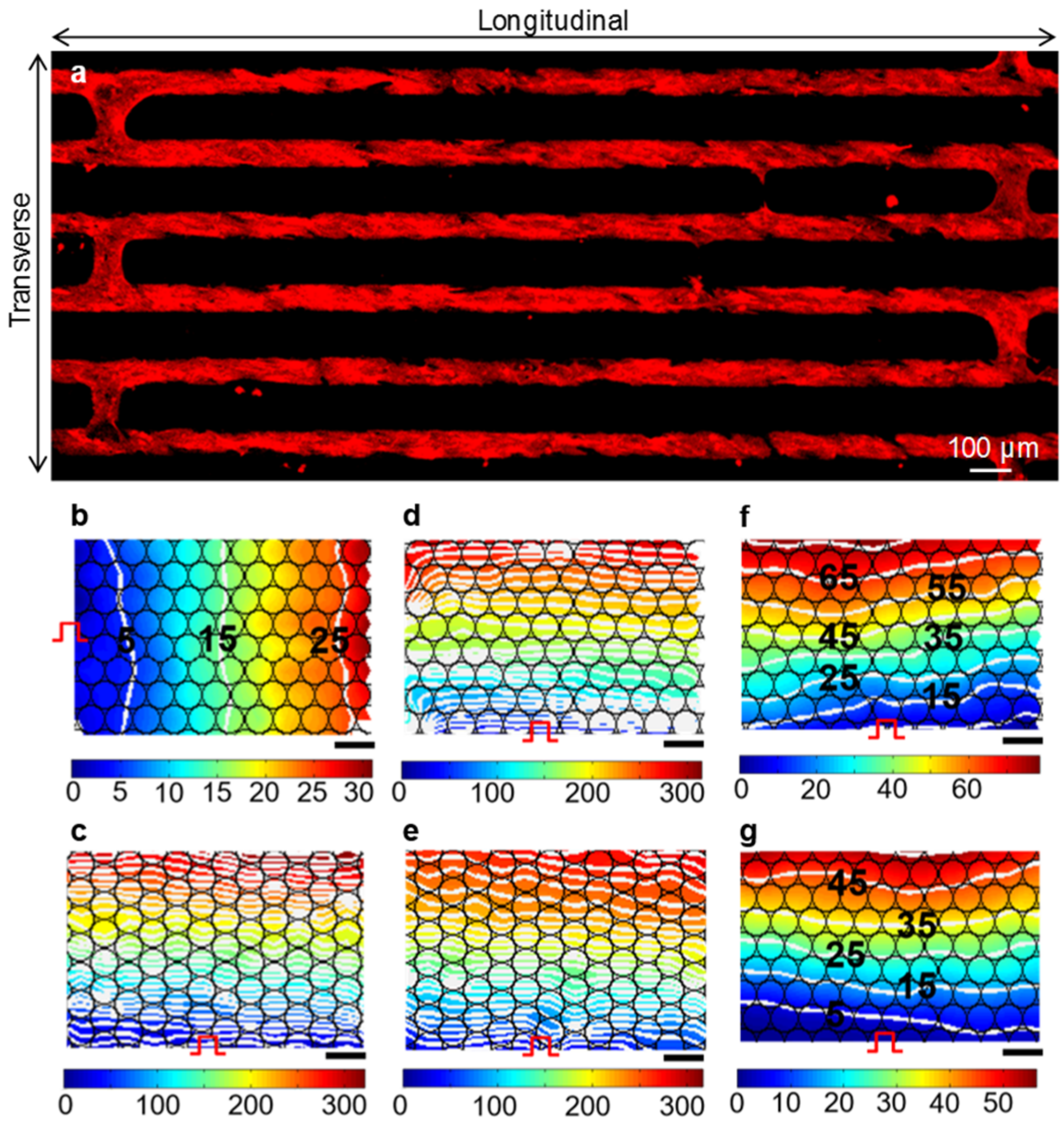

Supplementary Figure 9 | Use of E-HDFs to improve impaired cardiomyocyte conduction. (**a**) A zig-zag pattern of neonatal rat cardiomyocytes (CMs) used to simulate fast longitudinal and slow transverse AP conduction characteristic of cardiac tissue with interstitial fibrosis. Cells were stained with α-sarcomeric actin (red). (**b**,**c**) Representative fast longitudinal conduction (**b**) and slow transverse conduction (**c**) in CM monoculture. (**d**,**e**) Slow unaltered transverse conduction in CM + wt HDF co-culture (**d**) and CM + Na_vShePD60A+Kir2.1 HDF co-culture (**e**). (**f**,**g**) Moderately and significantly improved transverse conduction in CM + Cx43 HDF (**f**) and CM + E-HDF (**g**) co-cultures, respectively. In **b-g:** Scale bars, 1 mm. Pulse signs indicate positions of line stimulation. Circles denote recording sites.

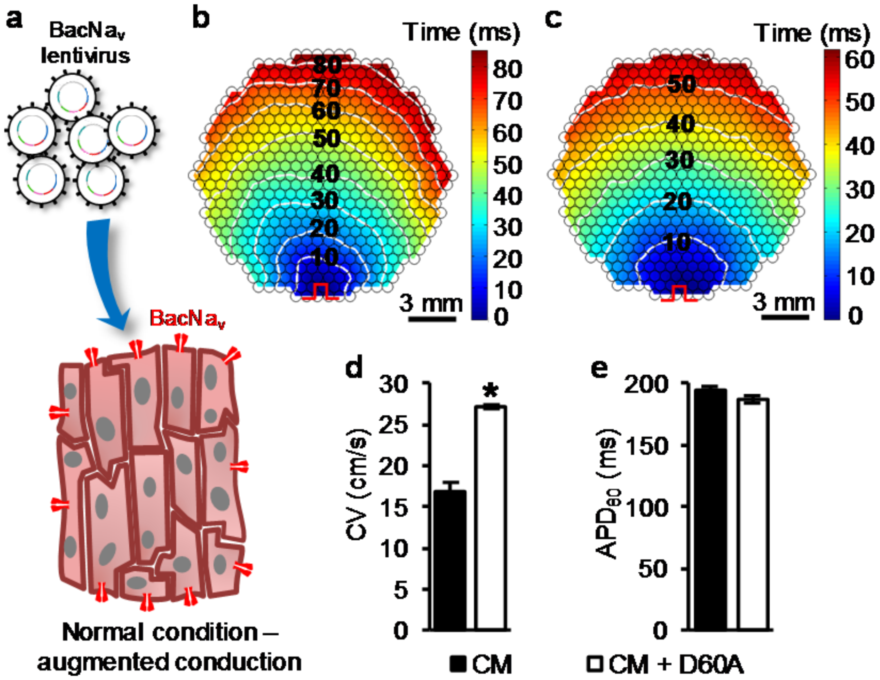

Supplementary Figure 10 | Enhancement of cardiac AP conduction by BacNav expression. (**a**) Schematic depicting exogenous expression of BacNa$_v$ in cardiomyocytes (CMs) to augment conduction. (**b**,**c**) Representative isochrone maps of AP conduction in electrically stimulated isotropic monolayers of control neonatal rat CMs (**b**) and CMs transduced with Na$_v$SheP D60A lentivirus (**c**). (**d**,**e**) CM monolayers transduced with Na$_v$SheP D60A lentivirus (CM + D60A) show increased CV (**d**) and unaltered APD$_{80}$ (**e**) compared to control CM monolayers (n = 5). *P<0.001 vs control CM. Pulse signs indicate location of stimulating electrode. Circles denote 504 recording sites. Error bars indicate s.e.m; statistical significance was determined by an unpaired Student's t-test to calculate P value.

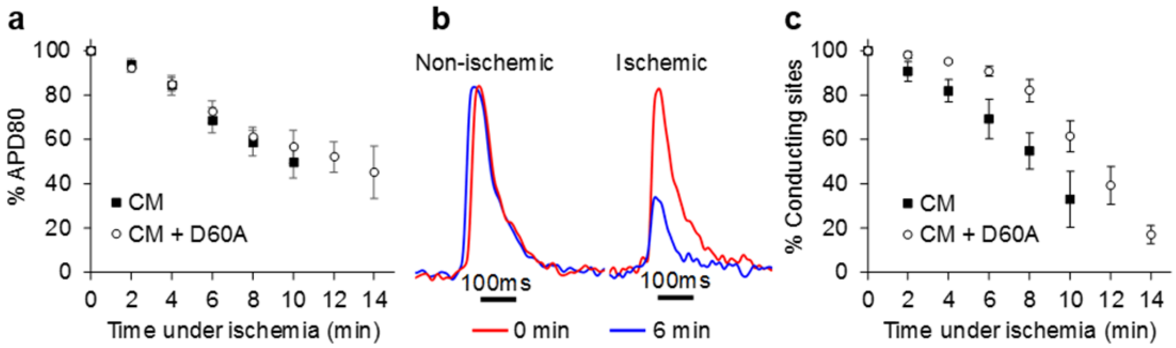

Supplementary Figure 11 | Consequences of ischemia on AP duration and firing in CM monolayers with regional ischemia. (**a**) With time of ischemia, AP duration gradually decreases in the ischemic region of monolayers made of either control cardiomyocytes (CM) or cardiomyocytes transduced with NavSheP D60A (CM + D60A) (n = 6). (**b**) Representative optical AP traces in the non-ischemic (peripheral) and ischemic (central) regions of the monolayer before (0 min) and during ischemia (6 min). (**c**) With time of ischemia, number of actively conducting recording sites within ischemic region is progressively reduced (n = 6). Error bars indicate s.e.m.

	Activation			Inactivation		
Channel	$V_{1/2}$ (mV)	Slope factor (mV)	Time constant (ms)	$V_{1/2}$ (mV)	Slope factor (mV)	Time constant (ms)
SheP WT	-36.7	8.1	1.6	-139.5	8.9	31.7
D60E	-39.6	7.4	2.0	-132.9	9.5	27.8
D60A	-7.6	7.1	3	-76.1	9	42.4
D60I	-9.5	6.8	19.2	-80.6	7.8	54
D60L	0.9	7.3	6.2	-81.9	10.1	57.3
D60V	14.2	8.2	4	-84.6	10.4	70.5
D60C	-6.5	7.3	7	-91.7	9.2	46.3
D60M	-8.1	7.6	6.2	-87.1	8.6	44.5
D60N	-9.3	6.2	4.2	-80.8	9.8	38.6
D60Q	0.5	7.7	5.2	-75.8	8.5	38.2
D60S	-5.5	7	2.4	-84.6	8.6	34.9
D60T	-1.8	6.8	6.2	-83.6	8.2	34.3
D60F	30.4	8.9	28.8	-55.4	8.6	193
D60W	8.9	11	11.4	-81.2	9.7	104.5
D60Y	1	8.7	16.5	-77.5	8.9	61.9
D60G	-1.5	6.6	7.5	-84.2	9.4	54.6
D60P	11.3	7.5	4	-71.4	10.5	51.6
D60H	4.3	6.9	9.3	-69	8	43.6
D60K	8.3	7.5	7.7	-63.4	7.5	44.3
D60R	8.4	6.4	4.8	-52.8	7.2	56.4
E43A	0.3	8.2	3.5	-57.7	13.2	53.1
E43L	2.9	8.2	2.4	-87.3	7.5	92
E43V	10	8.3	4	-106.4	10.5	40.2
E43N	0	8.2	2.6	-61.3	15.8	58.2
E43Q	25.1	9.2	2.5	-44.1	15.5	82.9
E43S	5.5	10	4.5	-58.7	14	23.6
E43T	-2.5	7.6	2.7	-68.8	4.5	51.8
E43H	16.7	11.3	4.4	-69.9	14.6	53.9
E43K	12.8	10.5	3	-50.3	11.2	24.1
RosD G217A	-16.6	10.5	17.5	-78.2	9.8	260.2

Supplementary Table 1 | Biophysical properties of BacNa$_v$ mutants. Activation and inactivation time constants were measured at +20mV clamp potential. Recordings were performed at 25^{0}C. Data shown are mean values (n = 4-10).

E43X	Primers	D60X	Primers
E43A	5'-ggctggctgaggtcgccatacctaactgc-3‘<br>5'-gcagttaggtatggcgacctcagccagcc-3'	D60A	5'-aaatacactcagtagcaacttagcaagactcatcaacaaagtacc-3‘<br>5'-ggtactttgttgatgagtcttgctaagttgctactgagtgtattt-3'
E43C	5'-catcaggctggctgaggtgcacatacctaactgcaccgc-3‘<br>5'-gcggtgcagttaggtatgtgcacctcagccagcctgatg-3	D60C	5'-cacaaatacactcagtagcaacttacaaagactcatcaacaaagtaccgtat-3‘<br>5'-atacggtactttgttgatgagtctttgtaagttgctactgagtgtatttgtg-3'
E43D	5'-ctggctgaggtatccatacctaactgcaccgcg-3‘<br>5'-cgcggtgcagttaggtatggatacctcagccag-3'	D60E	5'-gtactttgttgatgagtcttgagaagttgctactgagtgtatttg-3‘<br>5‘-caaatacactcagtagcaacttctcaagactcatcaacaaagtac-3'
E43F	5'-catcaggctggctgaggtgaacatacctaactgcaccgc-3‘<br>5'-gcggtgcagttaggtatgttcacctcagccagcctgatg-3'	D60F	5'-cacaaatacactcagtagcaacttaaaaagactcatcaacaaagtaccgtat-3‘<br>5'-atacggtactttgttgatgagtctttttaagttgctactgagtgtatttgtg-3'
E43G	5'-ggctggctgaggtccccatacctaactgc-3‘<br>5'-gcagttaggtatggggacctcagccagcc-3'	D60G	5'-aaatacactcagtagcaacttaccaagactcatcaacaaagtacc-3‘<br>5'-ggtactttgttgatgagtcttggtaagttgctactgagtgtattt-3'
E43H	5'-tcaggctggctgaggtatgcatacctaactgcacc-3‘<br>5'-ggtgcagttaggtatgcatacctcagccagcctga-3'	D60H	5'-atacactcagtagcaacttatgaagactcatcaacaaagtacc-3‘<br>5'-ggtactttgttgatgagtcttcataagttgctactgagtgtat-3'
E43I	5'-catcaggctggctgaggttatcatacctaactgcaccgc-3‘<br>5'-gcggtgcagttaggtatgataacctcagccagcctgatg-3'	D60I	5'-cacaaatacactcagtagcaacttaataagactcatcaacaaagtaccgtat-3‘<br>5'-atacggtactttgttgatgagtcttattaagttgctactgagtgtatttgtg-3'
E43K	5'-ggctggctgaggtcttcatacctaactgcac-3‘<br>5'-gtgcagttaggtatgaagacctcagccagcc-3'	D60K	5'-cactcagtagcaacttcttaagactcatcaacaaagtaccgtattgcgc-3‘<br>5'-gcgcaatacggtactttgttgatgagtcttaagaagttgctactgagtg-3'
E43L	5'-catcaggctggctgaggttaacatacctaactgcaccgc-3‘<br>5'-gcggtgcagttaggtatgttaacctcagccagcctgatg-3'	D60L	5'-cactcagtagcaactttaaaagactcatcaacaaagtaccgtattgcgc-3‘<br>5'-gcgcaatacggtactttgttgatgagtcttttaaagttgctactgagtg-3'
E43M	5'-caggctggctgaggtcatcatacctaactgcacc-3‘<br>5'-ggtgcagttaggtatgatgacctcagccagcctg-3'	D60M	5'-cactcagtagcaacttcataagactcatcaacaaagtaccgtattgcgc-3‘<br>5'-gcgcaatacggtactttgttgatgagtcttatgaagttgctactgagtg-3'
E43N	5'-atcaggctggctgaggtattcatacctaactgcaccg-3‘<br>5'-cggtgcagttaggtatgaatacctcagccagcctgat-3'	D60N	5'-aatacactcagtagcaacttattaagactcatcaacaaagtaccg-3‘<br>5'-cggtactttgttgatgagtcttaataagttgctactgagtgtatt-3'
E43P	5'-caggctggctgaggtcggcatacctaactgcacc-3‘<br>5'-ggtgcagttaggtatgccgacctcagccagcctg-3'	D60P	5'-caaatacactcagtagcaacttaggaagactcatcaacaaagtaccgt-3‘<br>5'-acggtactttgttgatgagtcttcctaagttgctactgagtgtatttg-3'
E43Q	5'-ggctggctgaggtctgcatacctaactgcac-3‘<br>5'-gtgcagttaggtatgcagacctcagccagcc-3'	D60Q	5'-actcagtagcaacttctgaagactcatcaacaaagtaccgtattgc-3‘<br>5'-gcaatacggtactttgttgatgagtcttcagaagttgctactgagt-3'
E43R	5'-caggctggctgaggtcctcatacctaactgcacc-3‘<br>5'-ggtgcagttaggtatgaggacctcagccagcctg-3'	D60R	5'-caaatacactcagtagcaacttacgaagactcatcaacaaagtaccgt-3‘<br>5'-acggtactttgttgatgagtcttcgtaagttgctactgagtgtatttg-3'
E43S	5'-caggctggctgaggtcgacatacctaactgcacc-3‘<br>5'-ggtgcagttaggtatgtcgacctcagccagcctg-3'	D60S	5'-cacaaatacactcagtagcaacttactaagactcatcaacaaagtaccgtat-3‘<br>5'-atacggtactttgttgatgagtcttagtaagttgctactgagtgtatttgtg-3'
E43T	5'-caggctggctgaggtcgtcatacctaactgcacc-3‘<br>5'-ggtgcagttaggtatgacgacctcagccagcctg-3'	D60T	5'-cacaaatacactcagtagcaacttagtaagactcatcaacaaagtaccgtat-3‘<br>5'-atacggtactttgttgatgagtcttactaagttgctactgagtgtatttgtg-3'
E43V	5'-ggctggctgaggtcaccatacctaactgc-3‘<br>5'-gcagttaggtatggtgacctcagccagcc-3'	D60V	5'-ggtactttgttgatgagtcttgttaagttgctactgagtgtattt-3‘<br>5'-aaatacactcagtagcaacttaacaagactcatcaacaaagtacc-3'
E43W	5'-caggctggctgaggtccacatacctaactgcacc-3‘<br>5'-ggtgcagttaggtatgtggacctcagccagcctg-3'	D60W	5'-cactcagtagcaacttccaaagactcatcaacaaagtaccgtattgcgc-3‘<br>5'-gcgcaatacggtactttgttgatgagtctttggaagttgctactgagtg-3'
E43Y	5'-atcaggctggctgaggtatacatacctaactgcaccg-3‘<br>5'-cggtgcagttaggtatgtatacctcagccagcctgat-3'	D60Y	5'-aatacactcagtagcaacttataaagactcatcaacaaagtaccg-3‘<br>5'-cggtactttgttgatgagtctttataagttgctactgagtgtatt-3'

Supplementary Table 2 | List of mutagenesis primers used to create Na_vSheP E43X and D60X libraries (designed by QuikChange® Primer Design Program).

**Ionic concentrations**	
Intracellular Na+ concentration	10.7 mM
Extracellular Na+ concentration	135.3 mM
Intracellular K+ concentration	120 mM
Extracellular K+ concentration	5.4 mM
**Membrane properties**	
Total membrane surface area (A)	2134 μm^2
Total membrane capacitance (C_m)	22 pF
BacNa$_v$ maximum conductance ($\overline{G}_{Na}$)	81.51 nS
K_{ir}2.1 maximum total conductance ($\overline{G}_{K1}$)	87.93 nS
Resting membrane potential (V_{rest})	-80.1 mV
Sodium Nernst potential (E_{Na}) at 37^{0}C	67.8 mV
Potassium Nernst potential (E_K) at 37^{0}C	-82.8 mV
**Core-conductor model parameters**	
Time step (Δt)	0.0025 ms
Space step (Δx)	24 μm
Cable radius	10.0 μm
Cable length	0.72 cm
Specific membrane capacitance (c_m)	1.031 $\mu F/cm^2$
Intracellular resistivity (R_i)	1.48 kΩ.cm

Supplementary Table 3 | Computational modeling parameters. Cell membrane properties (A, C_m, $\overline{G}_{Na}$, $\overline{G}_{K1}$, V_{rest}, E_{Na}, and E_K) used in the model were mean experimental values derived from whole-cell voltage-clamp recordings in a monoclonal HEK293 line expressing K_{ir}2.1, Cx43, and Na$_v$SheP D60A. Extracellular Na^+ and K^+ concentrations used in the model were the same as used in the bath solution during patch clamp and optical mapping experiments. Intracellular Na^+ and K^+ concentrations in the model were derived using Nernst equation, extracellular Na^+ and K^+ concentrations and E_{Na} and E_K, respectively. Specific membrane capacitance (c_m) used in the model was obtained by dividing mean cell membrane capacitance (C_m) with mean estimated cell membrane surface area (A). Intracellular resistivity (R_i) was adjusted to exactly simulate the mean conduction velocity (21.04 cm/s) recorded by optical mapping in confluent isotropic monolayers of the above-mentioned monoclonal HEK293 line expressing K_{ir}2.1, Cx43, and Na$_v$SheP D60A.
